# Gestational weight gain among pregnant women in Ibadan, Nigeria: Pattern, predictors and pregnancy outcomes

**DOI:** 10.1371/journal.pone.0290102

**Published:** 2023-08-18

**Authors:** Ikeola A. Adeoye, Elijah A. Bamgboye, Akinyinka O. Omigbodun

**Affiliations:** 1 Faculty of Public Health, Department of Epidemiology and Medical Statistics, College of Medicine, University of Ibadan, Ibadan, Nigeria; 2 Consortium of Advanced Research for Africa (CARTA), Nairobi, Kenya; 3 Faculty of Clinical Sciences, Department Obstetrics and Gynaecology, College of Medicine, University of Ibadan, Ibadan, Nigeria; University of Peradeniya, SRI LANKA

## Abstract

**Background:**

Gestational weight gain (GWG) is a risk factor for adverse pregnancy outcomes, future obesity and chronic diseases among women. However, has not received much attention in many low and middle-income countries such as Nigeria. We investigated the pattern, associated factors and pregnancy outcomes of GWG in Ibadan, Nigeria, using the Ibadan Pregnancy Cohort Study (IbPCS).

**Methodology:**

The IbPCS is a multicentre prospective cohort study conducted among 1745 pregnant women recruited from four health facilities in Ibadan, Nigeria. GWG, the primary outcome, was categorised according to the Institute of Medicine’s classification into insufficient, adequate and excessive weight gain. Pregnancy outcomes were the secondary outcome variables. Logistic regression analysis (Adjusted odds ratios and 95% confidence interval CI) was used to examine associations, and Poisson regression analyses were used to investigate associations with outcomes.

**Results:**

Only 16.9% of women had optimal GWG, 56.8% had excessive GWG, and 26.9% had insufficient GWG. Excessive GWG was associated with high income ’> #20,000-’ (AOR: 1.64, 95% CI: 1.25–2.17), being overweight (AOR: 2.12, 95% CI: 1.52–2.95) and obese (AOR: 1.47, 95% CI: 1.02–2.13) after adjusting for confounders. In contrast, increased odds of insufficient GWG have associated women with depression (AOR: 1.70, 95% CI 1.17–2.47). There was no significant association between inappropriate GWG and pregnancy outcomes However, there was an increased odds for postpartum haemorrhage (AOR: 2.44, 95% CI 1.14–5.22) among women with obesity and excessive GWG.

**Conclusions:**

Excessive GWG was the most typical form of GWG among our study participants and was associated with high maternal income, and being overweight or obese. GWG needs to be monitored during antenatal care, and interventions that promote appropriate GWG should be implemented among pregnant women in Nigeria.

## Introduction

Gestational weight gain (GWG) is the weight gained during pregnancy, a physiologic response necessary for foetal growth and development and the increased metabolic demand of pregnancy [1]. Inappropriate GWG is a public health concern associated with adverse pregnancy outcomes, future obesity and chronic diseases among women. The 2009 Institute of Medicine (IOM) guideline provided specific recommendations for GWG according to BMI based on the WHO classification [2]. Precisely, underweight women, normal weight, overweight and obese are to achieve a recommended total weight gain as follows (12.5–18.0) kg, (11.5–16.0) kg, (7.0–11.0) kg, (5.0–8.0) kg respectively. Also, the recommended weight gain per week in the second and third trimesters in kg/week are underweight (0.44–0.58), normal weight (0.35–0.50), overweight (0.23–0.33) and obese (0.17–0.27) [3,4]. Therefore women who gain weight below the recommended threshold have insufficient GWG, which is associated with intrauterine growth retardation, low birth weight, prematurity, and an increased risk of neonatal morbidity and mortality [5,6]. Women who gained weight within the stipulated range are described as having adequate GWG, while those that exceed the recommendation have excessive GWG. Excessive GWG adversely affects maternal metabolism and foetal development and is associated with preeclampsia, gestational diabetes mellitus, caesarean section and macrosomia [7–10]. Its long-term complications include postpartum weight retention and perpetuating the cycle of obesity among women of reproductive age [7,11,12].

Appropriate weight gain is crucial for optimal pregnancy outcomes, and weight gained outside the recommended range and the associated adverse health outcomes will increase the health care cost, the length of hospital stay, maternal morbidity and mortality [13]. In the past, insufficient GWG had been the primary concern among pregnant women in low and middle-income countries (LMIC) [3,7]. However, the current global obesity epidemic among women of reproductive age, which has resulted from urbanisation, globalisation, and changes in food consumption patterns and physical inactivity, has also led to excessive GWG from excess caloric intake [14]. Various inappropriate GWGs have been reported in different countries, mainly from North America and Europe, some from Asia. At the same time, evidence is sparse in Africa, especially those that used the IOM classification. For example, a systematic review and meta-analysis of over one million pregnant women from 23 studies from North America and Europe reported that 23% and 47% had insufficient and excessive GWG, respectively. Other countries have also documented the prevalence of insufficient and excessive GWG, namely China (22.6% versus 50%) [15]; Brazil (22.6% versus 50%) [16]; Turkey (10.8% versus 14.0%) [17]; Cameroun (40% versus 32%) [18]. Notably, a systematic review that assessed GWG in Africa from 26 studies noted that insufficient GWG was predominant in most African countries, particularly the poorest countries [19]. In contrast, South Africa had the highest proportion of excessive GWG (55%) in the region. The predictors of GWG in Africa include maternal education, income, obtaining a minimum of four ANC visits, being physically active, revenue, and dietary pattern [19,20].

Nigeria bears a considerable burden of maternal mortality with a maternal mortality ratio of 578 maternal deaths per life birth and contributes 12% to global figures annually (35 000 maternal deaths [21]. At the same time, Nigeria is undergoing nutrition and epidemiological transitions, leading to an upsurge of obesity in the general population, including women of reproductive age [22]. The coexistence of obesity and excessive GWG leads to more severe pregnancy complications. For instance, studies among Chinese women showed that obese women with excessive GWG had much higher odds for hypertensive disorders of pregnancy than normal-weight women with adequate weight gain [23–25]. Despite this, GWG has been a neglected maternal health issue in Nigeria. Although assessing maternal weight at every antenatal visit is routine, GWG is neither evaluated nor addressed in most maternal healthcare settings in Nigeria [26,27]. This contrasts with the practice in the United States, where The American College of Obstetricians and Gynaecologists (ACOG) recommends that *"Maternal care workers*, *which include obstetricians*, *doctors*, *midwives and nurses should assess women’s BMI at the antenatal booking visit*. *They also provide appropriate information*, *education and communication on maternal nutrition and exercise*. *Optimal weight and the importance of preventing excessive weight gain* [28]. Furthermore, the current body of evidence on GWG in Nigeria is limited, with only a few emerging studies available [29–32]. Asefa *et al*. (2020) noted a lack of research, the poor quality and methodology associated with GWG studies in Africa, hence suggesting the need for methodologically sound studies to address GWG-related research questions, including the related factors and pregnancy outcomes in sub-Saharan Africa [19]. Therefore we investigated the pattern, prevalence, predictors and pregnancy outcomes of GWG in Ibadan, Nigeria, using the Ibadan pregnancy cohort study.

## Materials and methods

### Study design, setting and population

This study was a component of the Ibadan Pregnancy Cohort Study (IbPCS), which aimed to investigate the associations between maternal obesity, lifestyle characteristics, glycaemic control, GWG and the pregnancy outcomes in Ibadan. The details of the methodology have been published elsewhere [33]. In summary, IbPCS is a prospective cohort study conducted among 1745 pregnant women in Ibadan, Nigeria, who were recruited early in pregnancy (GA ≤ 20 weeks) at their first antenatal visit at four selected health facilities in Ibadan. These facilities were University College Hospital, Adeoyo Maternity Teaching Hospital, Jericho Specialist Hospital, and Saint Mary Catholic Hospital, Oluyoro Ibadan. They provide comprehensive obstetric services to pregnant women and are the major referral centres for obstetric emergencies within the Ibadan metropolis. During recruitment, participants were assessed by trained research nurses and data were obtained on baseline information and lifestyle characteristics through personal interviews and a desktop review of medical records. The baseline information included sociodemographic characteristics, obstetric and past medical history, and specific lifestyle characteristics.

**Fig 1:**
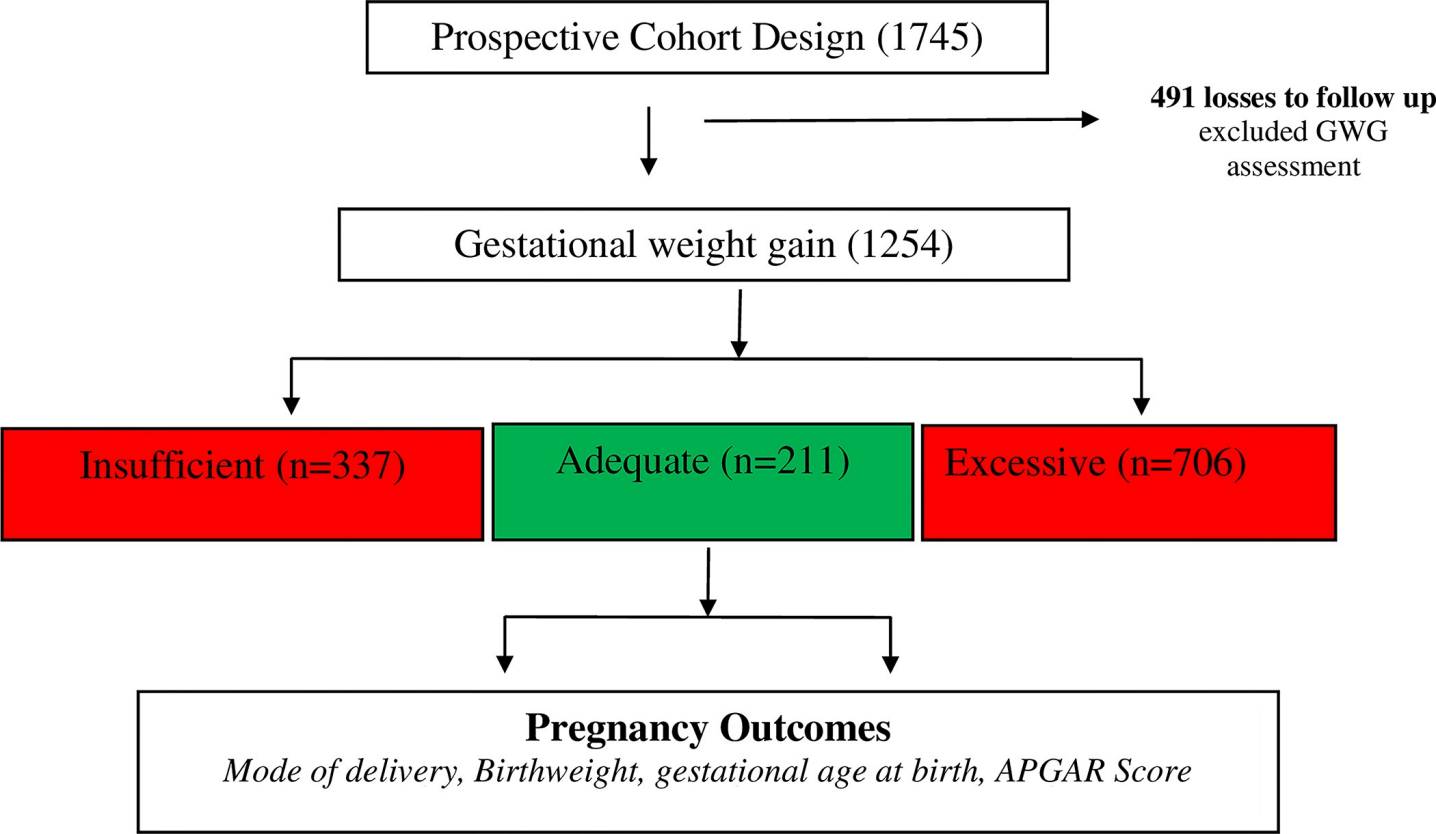
Flowchart of study participants.

The lifestyle factors examined included dietary patterns, including sugar-sweetened beverages (SSB), physical activity, sedentary behaviour, tobacco use, alcohol consumption, and sleep pattern. Anthropometric measures like weight, height and mid-upper arm circumference were obtained. Serial weight values were used to estimate GWG according to the Institute of Medicine guidelines (2009) [4] which was the difference between the booking weight and the last weight assessed during the third trimester at the ANC (32.4 ± 4.7 weeks). After recruitment (≤ 20 weeks), study participants were followed up at three time points: 24–28 weeks, third trimester, at delivery. The flowchart of study participants from enrolment to delivery is shown in Fig 1 and 1254 out of the 1745 participants were included in the final analysis.

#### Ethical approval

The **e**thical approval for this study was obtained from the University of Ibadan/University College Hospital (UI/UCH) Institutional Review Board (UI/EC/15/0060) and the Oyo State Ministry of Health Ethical Committee (AD/13/479/710). We obtained verbal and written informed consent from respondents before recruitment into the study. The study protocol and conduct adhered to the principles in the Declaration of Helsinki. Minors (participants under the age of 18 years) were not included in this study.

### Data collection procedures

#### Measures

Data were collected by trained research staff using pretested, interviewer-administered questionnaires and desktop review of medical records at booking, third trimester, and delivery. Ideally, GWG measures the difference between the weight at delivery and the prepregnancy weight. However, these were unavailable; hence the difference between the booking weight and the last third-trimester weight measured was used to estimate the GWG. The GWG rate was calculated as the difference between the last third-trimester weight recorded and the booking weight divided by the number of gestational weeks between in the interval these weights were measured as annotated as follows.

$$GWG\;rate\;=\frac{third\;trimester\;weight\;\left(final\right)-\;weight\;at\;booking}{GA\;at\;third\;trimester\;weight\;\left(final\right)-\;GA\;at\;booking}$$

The Institute of Medicine (IOM) guidelines provide recommendations for weight gain during pregnancy based on a woman’s pre-pregnancy body mass index (BMI). Hence, we categorised the GWG rate into insufficient, adequate, and excessive weight gain. Insufficient GWG was defined as below the lower BMI recommended weight gain per week, and excessive GWG as above the upper BMI recommended weight gain per week [1]. During the antenatal booking visit, body mass index (BMI) was estimated as a function of weight in kilograms per height in meter square. BMI was categorised by WHO classification: Underweight<18.5 kg/m^2^, Normal (healthy weight) = 18.5–24.9 kg/m^2^, overweight = 25.0–29.9 kg/m^2^, obesity≥ 30 kg/m^2^ [34]. The early pregnancy BMI obtained at booking was used as a proxy for prepregnancy BMI.

#### Exposure variables

These included maternal age (< 35 years, ≥ 35 years), parity (nulliparous, 1–3, ≥ 4), marital status (single or married), level of education (primary, secondary or tertiary), employment status (employed, unemployed), religion (Christianity, Islam), ethnicity (Yoruba versus non Yoruba), average monthly income level (< 20,000, 20,000–99,999; ≥ 100,000), Lifestyle characteristics such as alcohol consumption (yes or no), and tobacco exposure (yes or no), BMI, SSB intake (low/high). The participants’ level of physical activity is assesses using the (i.) passive transport (ownership of motorized transport) i.e. physically inactive while active transport or walking as physically active) [35,36] (ii.) Duration of moderate-intensity exercise (minutes per week) is the total time spent on moderate-intensity activity per week [37]. (iii.) Sedentary behaviour was also assessed using the pregnancy physical activity questionnaire (PPAQ) and classified as high or low according to the PPAQ instruction guide [38,39]. Psychological factors such as antepartum depression ascertained using the Edinburgh Postnatal Depression Scale (EPDS) score ≥ 12 [40]. Health care utilization assessed by the number of ANC visits (< 4 or ≥4) as well as a history of chronic medical illness (yes or no).

### Pregnancy outcomes

The pregnancy outcomes assessed included gestational diabetes mellitus (GDM), mode of delivery, birth weight, gestational age at birth and birth asphyxia at 1 and 5 minutes. *Gestational Diabetes Mellitus*: GDM diagnosis was made based on the International Association of Diabetic and Pregnancy Study Group (IADPSG) criteria in which GDM was present if one of the thresholds FPG ≥ 5.1 mmol/l; 1-hour plasma glucose ≥ 10.0 mmol/l, 1-hour plasma glucose ≥ 8.5 mmol/l was surpassed. GDM was diagnosed based on a 75-g 2-hour oral glucose tolerance test (OGTT) at pregnancy 24–28 weeks [19]. *Mode of delivery* included spontaneous vaginal delivery (SVD), Caesarean section (CS), and Induction of labour (IOL). *Birth weight*: The birth weight of the infant at birth is grouped into Low Birth Weight (< 2500g), Normal weight (2500 – 3999g) Macrosomia (≥ 4000g) [41]. *Gestational age at delivery*: This included Preterm (< 37 weeks), Term (37–42 weeks) Postdate (> 42 weeks) [42]. *APGAR score*: Good (≥ 7) or Birth asphyxia (< 7) [43]. v.) *Postpartum Haemorrhage*: Blood loss ≥ 500 MLS post-vaginal delivery and ≥ 1000mls post-caesarean section [42,44].

### Statistical analyses

Statistical analyses were performed using STATA version 13 software for Windows (Stata Corp TX). The study participants were divided according to the category of GWG: insufficient, adequate, and excessive. The characteristics of pregnant women, namely sociodemographic, lifestyle, psychosocial, and maternal care utilisation, were assessed across the categories of GWG using the chi-square test. The dependent variables were insufficient and excessive GWG (adequate GWG was the reference category) and pregnancy outcomes. The factors associated with insufficient and excessive GWG were assessed using binary logistic regression, and we reported the Odds Ratio and 95% confidence interval. The factors investigated were maternal age, employment status, level of education, ethnicity, religion, monthly income, parity, body mass index, SSB intake, physical activity levels, duration of sleep, tobacco exposure, and alcohol consumption during pregnancy, depression, history of chronic medical disease, and the number of antenatal care visit. Variables significant at a 5% level of statistical significance at binary logistic analysis were subjected to multiple logistic regression analyses (religion, income, BMI, physical activity level, and APD). The estimated unadjusted and adjusted odds ratios, 95% confidence intervals and p-values (p<0.05) of associated factors were reported.

The incidence proportion of pregnancy outcomes by GWG was also assessed and reported. Poisson regression analysis was used to determine the association between GWG and pregnancy outcomes. Incidence risk ratio (relative risk); 95% CI and p-values were reported. Forest plots showing the association of specific pregnancy outcomes with GWG based on maternal BMI” were also presented.

## Results

### Participants’ characteristics according to GWG

The characteristics of the study participants according to the GWG are shown in Table 1. The mean age of study participants was 29.8 ± 5.3 years and majority belonged to the Yoruba ethnic group (88.8%). The prevalence of GWG across the three categories were insufficient 337 (26.9%), adequate 211 (16.8%), and excessive 706 (56.3%) GWG. GWG was associated with maternal education (p<0.001), religion (p<0.001), monthly income (p<0.001), physical activity (p = 0.005), BMI (p<0.001), and antepartum depression (p<0.001). Specifically, the level of education and income had a positive association with excessive GWG i.e. women with primary education or less: 15 (45.5%), secondary 151 (44.0%), and tertiary 537 (61.4%) and income: *< 20*,*000*–192 (46.7%), *20*,*000–99*,*999*, 373 (63.2%), and *≥ 100*,*000*, 48 (61.5%). Also, more Christians (60.7%) than Muslims (49.6%) experienced excessive GWG. Women excessive GWG spent significantly less time on moderate intensity activity per week (25.3±23.1) minutes, more physically inactive (60.7%) and more sedentary (61.0) than women with inadequate GWG who spent (28.4 ± 23.6) minutes/week on moderate intensity activity, less more physically inactive (52.0%) and less sedentary (52.3%). Conversely, women who experienced antepartum depression (APD) had a higher proportion of insufficient GWG (39.2%) compared to women without APD (24.9%).

**Table 1:** Characteristics of pregnant women by GWG in Ibadan, Nigeria.

		GWG			
	Total (N-1254)	Insufficient (n = 337)	Adequate (n = 211)	Excessive (n = 706)	p-value
Proportion		26.9%	16.8%	56.3%	
**Age group**					
< 35	1004 (80.1)	267(26.6)	176(17.5)	561(55.9)	0.409
≥ 35 years	250 (19.9)	70(28.0)	35 (14.0)	145(58.0)	
**Parity**					
Nulliparous	552(44.3)	134 (24.3)	102 (18.5)	316 (57.2)	0.126
1–3	636 (51.0)	180 (28.3)	102 (16.0)	354 (55.7)	
≥ 4	59 (4.7)	22 (37.3)	6 (10.2)	31 (52.5)	
**Marital Status**					
Single	67 (5.3)	24 (35.8)	6 (9.0)	37 (55.2)	0.094
Married	1187 (94.7)	313 (26.4)	205 (17.3)	669 (56.3)	
**Maternal Education**					
At least Primary	33 (2.6)	13 (39.4)	5 (15.1)	15 (45.5)	**<0.001**
Secondary	343 (27.5)	128 (37.3)	64 (18.7)	151 (44.0)	
Tertiary	894 (69.9)	196 (22.4)	141 (16.1)	537 (61.5)	
**Employment Status**					
Employed	1114 (88.8)	307 (27.6)	186 (16.7)	621 (55.7)	0.304
Unemployed	140 (11.2)	30 (21.4)	25 (17.9)	85 (60.7)	
**Religion**					
Christianity	733 (58.9)	159 (21.7)	129 (17.6)	445 (60.7)	**<0.001**
Islam	512 (41.1)	177 (34.6)	81 (15.8)	254 (49.6)	
**Ethnicity**					
Yorubas	1112 (88.8)	311 (28.0)	191 (17.2)	610 (54.8)	0.050
Non-Yorubas	140 (11.2)	26 (18.6)	20 (14.3)	94 (67.1)	
**The income per month (Naira)** *					
<20,000	419 (38.1)	148(36.0)	71(17.3)	192(46.7)	**<0.001**
20,000–99,999	604 (54.9)	134(22.1)	98(16.2)	373(63.2)	
≥ 100,000	78 (7.1)	14 (18.0)	16 (20.5)	48(61.5)	
***Lifestyle characteristics***					
**BMI**					
Underweight	36 (2.9)	9 (25.0)	7 (19.4)	20 (55.6)	**<0.001**
Normal weight	622 (30.7)	189 (30.4)	139 (22.3)	294 (47.3)	
Overweight	337 (27.5)	74 (22.0)	38 (11.2)	225 (66.8)	
Obese	232 (18.9)	65 (28.0)	27 (11.6)	140 (60.4)	
**SSB Intake**					
Low	605 (49.8)	151 (25.0)	96(15.9)	358(59.2)	0.153
High	611 (50.2)	175 (28.0)	108(17.7)	328(53.7)	
**Physical Activity**					
Physically active	635(50.6)	183(28.8)	121(19.2)	330(52.0)	0.005
Physically inactive	619(49.4)	154(24.8)	89(14.5)	376(60.7)	
**Sedentary Behaviour**					
Low	561 (52.9)	171 (30.5)	95 (16.9)	295 (52.3)	0.005
High	518 (48.1)	114 (22.0)	88 (17.0)	316 (61.0)	
**Moderate intensity activity Duration (minutes)**	25.8±22.7	28.4±23.6	23.5±19.6	25.3±23.1	0.033
**Sleep Duration (hours)**	8.00±1.69	8.13± 1.64	8.07± 1.83	7.91± 1.66	0.114
**Tobacco exposure**					
Yes	42 (3.4)	11 (26.2)	6 (14.3)	25 (59.5)	0.088
No	1212 (96.6)	326 (26.9)	205 (16.9)	681 (56.2)	
**Alcohol Consumption**					
Yes	164 (13.1)	40 (24.4)	28 (17.1)	96 (59.5)	0.738
No	1090 (86.9)	297 (27.2)	183 (16.8)	610 (56.0)	
***Psychosocial factors***					
**Antepartum depression**					
Yes	176 (14.6)	69 (39.2)	29 (16.5)	78 (44.3)	**<0.001**
No	1025 (85.4)	255 (24.9)	173 (16.9)	597 (58.2)	
***Health care Utilisation***					
**ANC visit**					
<4visit	198 (25.1)	50 (25.2)	33 (16.7)	115 (58.1)	0.684
≥ 4 visits	590 (74.9)	133 (22.5)	95 (16.1)	362 (61.4)	
**Chronic medical disease**					
Yes	133 (10.6)	42 (31.6)	22 (16.5)	69 (51.9)	0.417
No	1121 (89.4)	295 (26.3)	189 (16.9)	637 (56.8)	

*US Dollars equivalent < 20,000 (45 USD), 99,999 (220 USD), ≥ 100,000 (225 USD).

### Gestational weight by maternal body mass index

**Fig 2:**
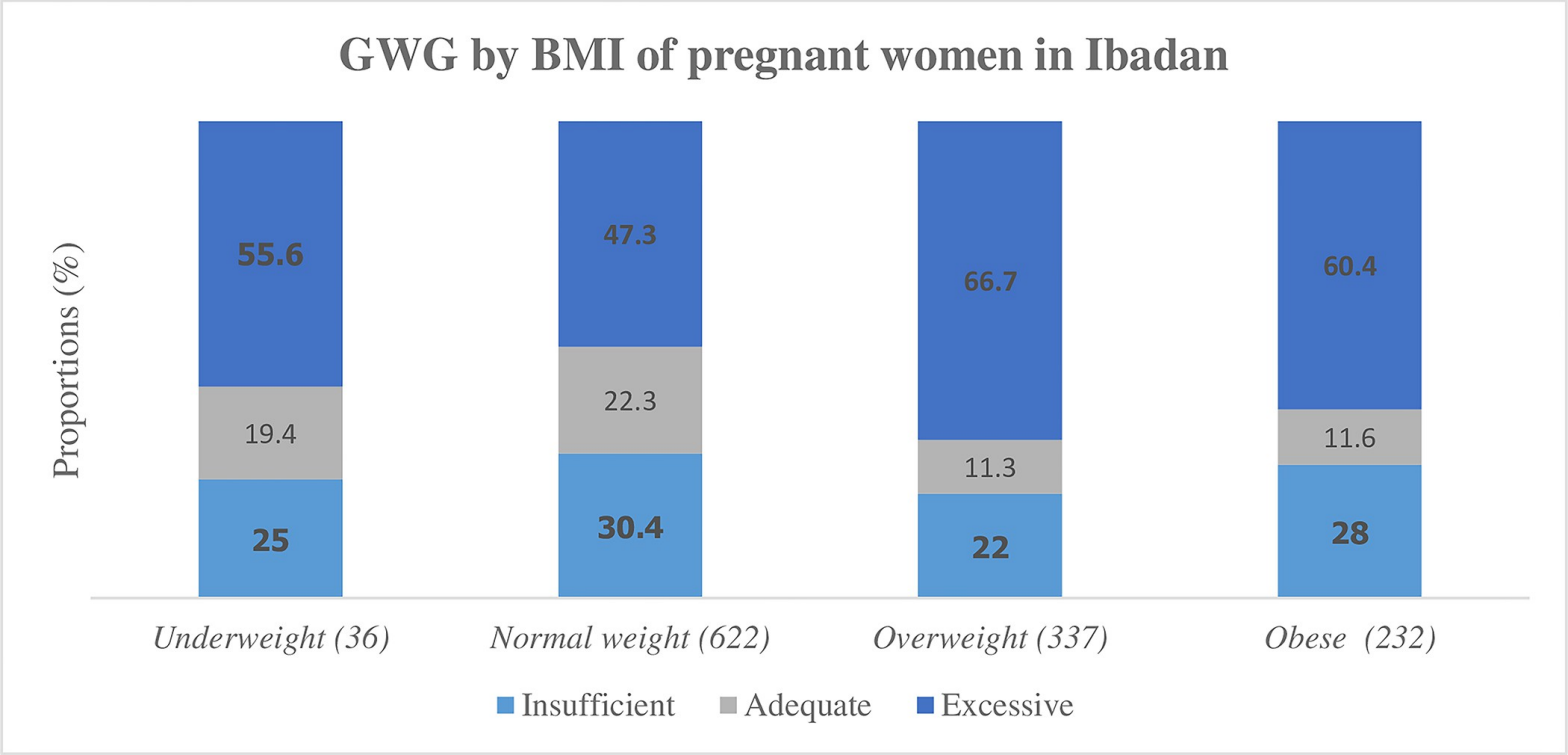
GWG according to BMI in Ibadan, Nigeria.

The pattern of GWG across maternal BMI is shown in Fig 2. The distribution of maternal BMI among the participants with GWG ***assessment*** was underweight 36 (3.0%), normal weight 622 (50.7%), overweight 337 (27.5%), and obese 232 (18.9%). Excessive GWG was the most familiar pattern of weight gain among the study participants across maternal BMI: underweight (55.6%), normal weight (47.3%), overweight (66.8%), and obese (60.4%). We observed adequate GWG was the least common.

### Factors associated with insufficient and excessive GWG among pregnant women in Ibadan, Nigeria

The factors associated with insufficient and excessive GWG are represented in Table 2. After adjusting for confounders, the factors related to GWG were maternal BMI, religion, income, level of physical activity and APD. Specifically, the odds of excessive GWG was associated with increasing revenue’ 20,000–99,999’ (AOR: 1.64, 95% CI: 1.25–2.17), ’≥ 100,000’ (AOR: 1.54, 95 CI: 0.90–2.64), compared to those who earned less than 20,000. Compared with women with normal weight, women with overweight (AOR: 2.07, 95% CI 1.52–2.88) and obese (AOR: 1.56, 95% CI 1.11–2.20) had a higher odds of excessive weight gain. Also women who had depression during pregnancy were also less likely to experience excessive GWG (AOR: 0.56, 95% CI 0.36–0.82). Conversely, compared with Christian women, Muslims had higher odds for insufficient GWG (AOR: 1.52, 95% CI 1.13–2.05). And women with APD had 70% higher odds of insufficient GWG (AOR: 1.70, 95% CI 1.17–2.45) than women without APD.

**Table 2:** Factors associated with insufficient and excessive GWG among pregnant women in Ibadan.

	Insufficient GWG		Excessive GWG	
	Unadjusted OR (95%CI)	Adjusted OR (95% CI)	Unadjusted OR (95% CI)	Adjusted OR (95% CI)
**Age group**				
Less than 35	1.00		1.00	
≥ 35 years	1.07 (0.79–1.46)		1.09 (0.82–1.44)	
**Employment**				
Unemployed	1.00		1.00	
Employed	1.40 (0.91–2.13)		0.82 (0.57–1.17)	
**Education**				
≤ Primary	1.00	1.00	1.00	
Secondary	0.92 (0.44–1.90)	1.13(0.49–2.55)	0.94 (0.46–1.93)	
Tertiary	0.45 (0.22–0.91)**	0.78 (0.35–1.73)	1.91 (0.95–3.85)	
**Ethnicity**				
Non Yorubas	1.00		1.00	
Yorubas	1.70 (1.09–2.66)		0.60 (0.41–0.86)	
**Religion**				
Christian	1.00	1.00	1.00	1.00
Islam	1.91 (1.48–2.46)**	1.52 (1.12–2.08)**	0.64 (0.51–0.80)**	0.73 (0.55–0.97)**
**Income**				
< 20,000	1.00	1.00	1.00	1.00
20,000–99,999	0.51 (0.39–0.68)**	0.53(0.41–0.76)**	1.90 (1.47–2.44)**	1.76 (1.31–2.37)**
≥ 100,000	0.39 (0.21–0.72)**	0.36 (0.17–0.74)**	1.89 (1.15–3.10)**	1.51 (0.85–2.70)
**Parity**				
Nulliparous	1.00		1.00	
1–3	1.23 (0.95–1.60)		0.94 (0.75–1.18)	
≥ 4	1.86 (1.06–3.26)		0.83 (0.48–1.42)	
**BMI**				
Underweight	0.76 (0.35–1.66)	0.53 (0.21–1.38)	1.40 (0.71–2.74)	2.02 (0.89–4.53)
Normal weight	1.00	1.00	1	1
Overweight	0.65 (0.47–0.88)**	0.69 (0.48–0.99)**	2.07 (1.52–2.88)**	2.12 (1.52–2.95)**
Obese	0.89 (0.64–1.25)	0.98 (0.66–1.45)	1.70 (1.25–2.31)**	1.47 (1.02–2.13)**
**SSB Intake**				
Low	1.00		1.00	1.00
High	1.20 (0.93–1.56)		0.80 (0.63–1.00)*	0.78 (0.57–1.06)
**Physical Activity**				
Physically active	1.00		1.00	1.00
Physically inactive	0.82(0.63–1.06)		143(1.14–1.79)**	1.25 (0.94–1.65)
**Sedentary Behaviour**				
Low	1.00		1	
High	0.64 (0.49–0.85)**	0.75 (0.55–1.02)	1.41 (1.11–1.80)**	1.26(0.96–1.67)
**Moderate intensity activity Duration (minutes)**	1.07 (0.99–1.12)		0.99 (0.99–1.002)	
**Sleep Duration**	1.01 (1.001–1.01		0.93 (0.87–0.99)**	0.95 (0.88–1.04)
**Tobacco exposure**				
Yes	0.96 (0.48–1.94)		1.15 (0.61–2.15)	-
No	1.00		1.00	-
**Alcohol Consumption**				
Yes	0.86 (0.59–1.26)		1.11 (0.80–1.55)	-
No	1.00		1.00	-
**Antepartum depression**				
Yes	1.95 (1.39–2.72)**	1.79 (1.21–2.65)**	0.57 (0.41–0.79)**	0.53 (0.36–0.79)**
No	1.00	1.00	1.00	
**ANC visits**				
<4visit	1.00		1.00	-
≥ 4 visits	0.86 (0.59–1.25)		1.15 (0.83–1.59)	-
**Chronic medical condition**				
Yes	1.29 (0.88–1.91)		0.82 (0.57–1.17)	-
No	1.00		1.00	-

### Pregnancy outcomes associated with GWG

The incidence proportions, relative risk and 95% CI of the pregnancy outcomes are shown in Table 3. The incidence of caesarean section increased with GWG: insufficient GWG (25.3%), adequate (29.3%): an excessive (36.7%). Also, women with excessive GWG had a 26% higher risk of undergoing a caesarean section: [unadjusted RR 1.26 (95% CI) (0.94–1.68); p-value: 0.129] compared to adequate GWG. Conversely, the incidence of SVD decreased with GWG, with excessive GWG having a 24% lower risk of SVD: [unadjusted RR 0.86 (95% CI) (0.70–1.05); p value< 0.145]. Excessive GWG was also associated with an increased risk of postpartum haemorrhage [unadjusted RR 1.24 (95% CI) (0.81–1.89); p value< 0.318]. There were no statistically significant associations observed between pregnancy outcomes and gestational weight gain.

**Table 3:** Association between GWG and pregnancy outcomes in Ibadan, Nigeria.

Pregnancy Outcomes	n/N	Incidence (%)	Unadjusted Relative Risk 95% CI	P-value
**Caesarean section**				
*Insufficient*	74/234	25.3	0.87 (0.61–1.23)	0.420
*Adequate*	55/188	29.3	1.00	
*Excessive*	234/637	36.7	1.26 (0.94–1.68)	0.129
**Spontaneous vaginal delivery**				
*Insufficient*	206/293	70.3	1.07 (0.85–1.33)	0.589
*Adequate*	127/192	66.2	1.00	
*Excessive*	366/643	56.9	0.86 (0.70–1.05)	0.145
**Induction of Labour**				
*Insufficient*	5/293	1.7	1.40 (0.13–1.25)	0.117
*Adequate*	8/192	4.2	1.00	
*Excessive*	29/643	4.5	1.08 (0.49–2.37)	0.843
**Macrosomia**				
*Insufficient*	14/236	5.6	1.39 (0.56–3.44)	0.475
*Adequate*	7/174	4.0	1.00	
*Excessive*	36/589	6.1	1.51 (0.68–3.41)	0.311
**Low birth weight**				
*Insufficient*	16/250	6.2	1.01 (0.47–2.18)	
*Adequate*	11/174	6.3	1.00	
*Excessive*	48/589	8.2	1.28 (0.68–2.48)	0.447
**Preterm delivery**				
*Insufficient*	43/272	15.8	1.17 (0.71–1.93)	0.532
*Adequate*	24/178	13.5	1.00	
*Excessive*	82/609	13.5	0.99 (0.63–157)	0.995
**Birth Asphyxia at 1 minute**				
*Insufficient*	22/165	13.3	0.71 (0.39–1.27)	0.248
*Adequate*	24/128	18.8	1.00	
*Excessive*	80/476	16.8	0.90(0.57–1.41)	0.638
**GDM**				
*Insufficient*	29/155	18.7	1.09 (0.62–1.91)	6.771
*Adequate*	21/122	17.2	1.00	
*Excessive*	73/340	21.5	1.25 (0.77–2.02)	0.372
**Postpartum Haemorrhage**				
*Insufficient*	33/293	11.3	0.80 (0.48–1.33)	0.392
*Adequate*	27/192	14.1	1.00	
*Excessive*	112/643	17.4	1.24 (0.81–1.89)	0.318

**Fig 3:**
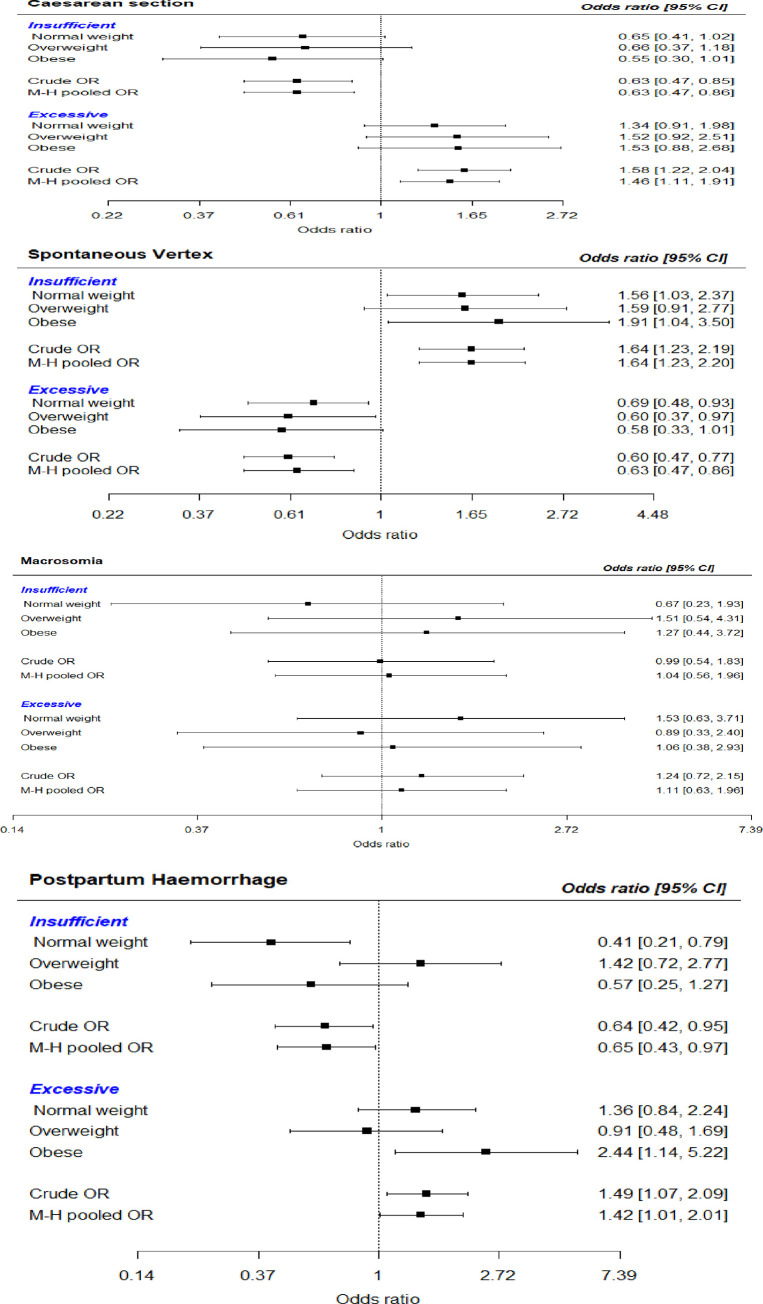
1_2_3_4: Forest plots showing the association of specific pregnancy outcomes with GWG by maternal BMI (caesarean section, spontaneous vaginal delivery, macrosomia and postpartum haemorrhage). Footnote for the forest plots ^1.^ Underweight weight was absent from the forest plots because they made up a very low proportion of the final analysis 36 (2.9%) and was mostly omitted from the output of the stratified analysis for the forest plots.

Forest plots showing the association of inadequate and excessive GWG with pregnancy outcomes (caesarean section, spontaneous vaginal delivery, macrosomia and postpartum haemorrhage) stratified by BMI are shown in Fig 3.

## Discussion

GWG is a neglected maternal health issue in Nigeria with a high maternal and neonatal health burden. Appropriate GWG is crucial for achieving optimal perinatal outcomes and preventing postpartum weight retention, future obesity and cardiometabolic complications. Hence optimal GWG contributes to health, well-being and sustainable development in women and children. Using the Ibadan Pregnancy Cohort study, we investigated the pattern, prevalence, predictors and outcomes of GWG among pregnant women in Ibadan. In this study, we assessed GWG using the rate of weight gain per week rather than the total weight gain, a more precise measure, This was because information required for the estimation total weight gain i.e. the weight just before the onset of labour and the pre-pregnancy weight, are not available in our study setting. Hence, in such a situation IOM guidelines recommends rate of weight gain per week assessed within the two and third trimester [2,4,8] which has also been put to use in other settings [4,8,6,45].

Importantly, we found that only 16.8% of our respondents achieved normal GWG, 26.9% had insufficient GWG, and more than half (56.3%) had excessive GWG. Excessive weight gain was more prevalent in our study population than insufficient GWG, a pattern commonly reported in high and middle-income countries like Canada, Australia and Brazil [15,16,46,47]. Conversely, low and middle-income countries have reported a higher prevalence of inadequate GWG than excessive [13,15,16,47–49]. Excessive GWG is an emerging maternal health issue in transiting economies with increased adverse perinatal outcomes such as macrosomia, increased operative delivery, postpartum weight retention, and maternal obesity in the subsequent pregnancies and future risk of non-communicable diseases [7,10,24,25,50–52]. The prevalence of excessive GWG reported in Nigeria ranges from 10.5–29.4% [30,53]. For example, Senbanjo et al. (2021) reported a lower prevalence (11.1%) among women in a tertiary health facility in Lagos, Nigeria. The variation in prevalence may be due to the differences in the participants’ characteristics, including the sociodemographic and lifestyle factors, timing of the study, study design, built environment and stressors in their study setting [54]. However, these researchers also reported a much higher prevalence of 54.1% for insufficient GWG. A recent systematic review and meta-analysis (2020) on the pattern of GWG in sub-Saharan Africa documented that insufficient GWG was the most common form of GWG occurring in more than half of the population [19]. Nonetheless, they also observed that excessive GWG was predominant in rich African countries such as South Africa, Nigeria, Ghana and Kenya [19].

Notably, the health systems in sub-Saharan Africa have focussed on maternal undernutrition and insufficient GWG from inadequate food intake, poor dietary quality, recurrent infections and short inter-pregnancy intervals more frequently than excessive weight gain and its complications [55]. Therefore, Nigeria’s maternal health care system should implement interventions that promote appropriate GWG. These interventions encompass creating awareness on the appropriate gestational weight gain (GWG) range, encouraging a healthy lifestyle during pregnancy through healthy diet and physical activity, routine self-monitoring of weight, preventing infection, and providing information on healthy weight gain and monitoring GWG among pregnant women [48].

Investigating the risk factors associated with inappropriate GWG is essential for providing targeted interventions for addressing the factors that contribute to undesirable weight gain during pregnancy among Nigerian women. We examined various factors associated with GWG, including sociodemographic, lifestyle, psychological, and healthcare factors. However, after adjusting for confounders, we found maternal BMI, religion, income, and APD significant factors. High income had a positive relationship (AOR = 1.68) with excessive GWG, with women with higher earnings having a higher odds of gaining excess weight. The positive association between income or high socioeconomic status and weight gain, particularly in LMIC developing countries, have been established in the literature [56,57]. High income affords more significant resources for improved access to food and increased food intake, including processed foods and sweetened drinks.

Additionally, undue reliance on technological and automated devices promotes physical inactivity and sedentary behaviours, which boosts weight gain. Conversely, high socioeconomic status is associated with an inverse relationship with weight gain in high-income countries because of the increased health literacy of the complications related to excessive weight gain [56–58]. Paul et al. (2013) corroborated our finding by reporting that low-income earning women in the US gained more weight than high-income earning women because they engaged in more weight-promoting behaviours [59].

We also found that women who were overweight or obese had a higher odds of excessive GWG than women with normal weight. This finding contradicts the 2009 IOM GWG guideline, which recommends a narrow weight gain limit for women with obesity [2] to minimise the adverse pregnancy outcomes associated with adiposity [13]. This finding could imply a lack of awareness of IOM guidelines on GWG in our maternal care services. A Nigerian study corroborated our study by reporting that more than half of their respondents said a lack of awareness of the risk involved with inappropriate GWG. At the same time, 22.4% of respondents reported that their healthcare provider had recommended that the women not gain weight beyond a specific limit (12–12.5kg)—not based on the IOM guidelines [31]. The IOM guidelines are not yet in place, nor is there any consensus on GWG in Nigeria’s maternal care [27,31]. For instance, based on the IOM guidelines, the American College of Obstetricians and Gynaecologists recommends that clinicians calculate a woman’s prepregnancy BMI at the first antenatal care visit and educate her on the importance of appropriate GWG goals [28]. Adopting and implementing a GWG guideline in Nigeria’s maternal health services is essential. Although the IOM guideline is based on cut-off points from white women and is yet to be validated among Africans, the IOM guidelines are still the most utilised GWG guideline. Hence it could be helpful in Nigeria up until local guidelines are developed.

Antenatal care provides a platform for education, counselling and monitoring GWG and the short and long-term problems associated with inappropriate weight gain should be discussed during ANC visits. In our study, the number of antenatal care attendance did not influence GWG. Conversely, compared with Christians, Muslims had higher odds for insufficient GWG (AOR: 1.52). Perhaps, because the religion imposes certain food restrictions, for example, on the pork intake that Christians are free to eat. The Ramadan fast may also prevent excessive weight gain because of reduced food intake. A Turkish study demonstrated a low prevalence of excessive GWG, probably for the same reasons [17]. Also, women with APD had 70% higher odds of insufficient GWG than women without APD. The reduced appetite, weight loss, poor self-care practices, and inadequate nutrition experienced by women with APD could explain this finding [60,61].

Importantly, our study investigated the influence of maternal lifestyle characteristics–SSB intake, sleep pattern and physical on GWG among our study population. We assessed physical activity using active/passive transport [35], duration of moderate-intensity exercise [35,37] and sedentary behaviour [38,39]. Active transport is a measure of physical activity because it contributes to total physical activity and increases energy expenditure [35,36]. Women excessive GWG spent less time on moderate intensity activity per week minutes, more physically inactive and more sedentary than women with inadequate GWG. We found that physically in active and sedentary women had higher odds for excessive GWG than physically active women. Although, the relationship became insignificant on multivariate analysis. Importantly, the WHO recommends that pregnant women engage in at least 150 minutes of moderate-intensity physical activity during the week [37]. Researchers have reported poor compliance with the recommendation among Nigerian pregnant women [62,63]. Hence the need to actively promote physical activity among pregnant women to obtain benefits, including improving cardiovascular fitness, preventing excessive GWG and GDM, improving sleep quality and so on [64–66]. Although there was a significant relationship between sleep duration and GWG, women with excessive GWG had a shorter sleep duration than those with adequate and insufficient sleep. Researchers have found an association between sleep deprivation and weight gain [67]. Further studies should further explore this relationship among the Nigerian pregnant population. In addition, although our study did not find any significant relationship between SSB intake and GWG, SSBs have been associated with obesity, weight gain and excessive GWG in the literature [68–70] because of the high and easily absorbed sugar content that leads to high energy intake and weight gain [71].

Notably, we estimated the incidence and relative risks of pregnancy outcomes by GWG. We found that GWG had an association with the mode of delivery. Excessive GWG had a 26% higher risk for the caesarean section (the association was the same for emergency or elective caesarean section). Although there was no statistical significance, this does not diminish its clinical or public health relevance. Studies have shown that excessive GWG increases the risk of caesarean delivery [23,72,73]. Plausible reasons for this association include a higher odds of maternal complications and cephalo-pelvic disproportion arising from the presence of a big foetus. Excessive GWG is accompanied by excess metabolic fuels, i.e. high maternal glucose, free fatty acid, and amino acid concentrations that are passed to the growing foetus leading to increase foetal weight. Even though not statistically significant, excessive GWG was associated with a 51% and 24% higher risk of macrosomia and postpartum haemorrhage than women with adequate GWG. While some studies have shown a positive association between excessive weight gain and gestational diabetes mellitus, a few have shown no association [15,74,75]. The lack of association between GWG and GDM in our study is likely due to lifestyle interventions and weight control, which restrict weight gain among women with GDM who obtain maternal care from facilities that provide comprehensive care [76]. Maternal BMI and GWG synergise maternal outcomes as the coexistence of obesity and excessive GWG leads to more severe complications [8,50]. For instance, studies among Chinese women showed that obese women with excessive GWG have much higher odds for hypertensive disorders during pregnancy than normal-weight women with adequate weight gain [23–25]. We found that excessive weight gain significantly increased the risk of postpartum haemorrhage among women who were obese. The association between maternal obesity postpartum haemorrhage has been reported in the literature [42,77,78]. The plausible reasons include poor uterine contractility in obese women compared with non-obese [79], increase foetal weight that could lead to uterine atony or perineal tear, associate large placenta and placenta praevia [78]. Blomberg et al. in the cohort study among Swedish pregnancy reported that the risk of atonic uterine haemorrhage increased rapidly with BMI [80]. Therefore, extra vigilance is advised in the active management of the third stage of labour in women with obesity and excessive GWG [78,79].

This study contributes significantly to a critical gap in maternal health literature in Nigeria, in which GWG has been scantly examined. Our study has several strengths, including the use of a prospective cohort study design which allowed for the direct measurement of critical variables, especially serial maternal weights, obtained by trained staff during every antenatal visit. Notably, previous studies had primarily relied on retrospective studies or self-reports subject to recall or misclassification bias. The prospective study design also allowed the investigation of multiple risk factors and outcomes associated with GWG. We investigated the influence of a broader range of variables; sociodemographic, lifestyle (alcohol consumption, tobacco exposure, SSB intake, physical activity and sleep duration), healthcare utilisation and psychological factors such as APD, which were lacking in previous studies. The use of multiple health facilities also enhanced the generalizability of the study. However, our study also has limitations. First is the bias from losses to follow-up, which is typical of prospective cohort studies but was accounted for by assuming a 40% attrition in the sample size calculation. In this study, we could not estimate total GWG because prepregnancy and delivery weights were unavailable; hence, we used a weekly GWG rate. Also, the use of booking weight at ≤ 20 weeks (mean 16.2 ± 4.7 weeks) women would have experienced some weight gain could have potentially influenced gestational weight gain estimate. Therefore, GWG rate used in this study was a more appropriate measure as recommended by IOM guidelines [4].

### Conclusions

Excessive GWG was the commonest form of GWG in our study population, with a prevalence of 56.3%, but insufficient and adequate GWG were observed in 26.9% and 16.8% of the pregnant women, respectively. The significant predictors of GWG were income, maternal BMI, religion, physical activity and APD. Even though the incidences of caesarean section and postpartum haemorrhage were higher among women with excessive GWG compared with adequate and insufficient GWG, we did not find any significant association between excessive GWG and pregnancy outcomes. Maternal obesity modified the relationship between GWG and pregnancy outcomes. Achieving appropriate GWG should be prioritise in Nigeria’s maternal health care.

## References

[1] Rasmussen KM, Yaktine ALE. Weight Gain During Pregnancy: Reexamining the Guidelines. Institute of Medicine. In: Rasmussen KM, Yaktine AL, editors. Weight Gain During Pregnancy: Reexamining the Guidelines. Washington (DC): National Academies Press (US) Copyright © 2009, National Academy of Sciences.; 2009. 20669500

[2] Institute of M, National Research Council Committee to Reexamine IOMPWG. The National Academies Collection: Reports funded by National Institutes of Health. In: Rasmussen KM, Yaktine AL, editors. Weight Gain During Pregnancy: Reexamining the Guidelines. Washington (DC): National Academies Press (US) Copyright © 2009, National Academy of Sciences.; 2009. 20669500

[3] IOM Committee on Nutritional Status During P, Lactation. Nutrition During Pregnancy: Part I Weight Gain: Part II Nutrient Supplements. Washington (DC): National Academies Press (US) Copyright © 1990 by the National Academy of Sciences.; 1990.

[4] IOM. Weight Gain During Pregnancy: Reexamining the Guidelines. In: Rasmussen KM, Yaktine AL, editors. The National Academies Collection: Reports funded by National Institutes of Health. Washington (DC): National Academies Press (US) Copyright © 2009, National Academy of Sciences.; 2009. 20669500

[5] Wang L, Zhang X, Chen T, Tao J, Gao Y, Cai L, Chen H, Yu C. Association of Gestational Weight Gain With Infant Morbidity and Mortality in the United States. JAMA Network Open. 2021;4(12):e2141498–e. doi: 10.1001/jamanetworkopen.2021.41498 34967878 PMC8719246

[6] Davis RR, Hofferth SL, Shenassa ED. Gestational Weight Gain and Risk of Infant Death in the United States. American journal of public health. 2013;104(S1):S90–S5. doi: 10.2105/AJPH.2013.301425 24354832 PMC4011101

[7] Oken E, Taveras EM, Kleinman KP, Rich-Edwards JW, Gillman MW. Gestational weight gain and child adiposity at age 3 years. American journal of obstetrics and gynecology. 2007;196(4):322.e1–.e3228. doi: 10.1016/j.ajog.2006.11.027 17403405 PMC1899090

[8] Siega-Riz AM, Bodnar LM, Stotland NE, Stang. The Current Understanding of Gestational Weight Gain among Women with Obesity and the Need for Future Research. NAM Perspectives Discussion Paper,. National Academy of Medicine, Washington, DC. 2019. 10.31478/202001a PMC8406571 34532680

[9] Nelson SM, Matthews P, Poston L. Maternal metabolism and obesity: modifiable determinants of pregnancy outcome. Human reproduction update. 2010;16(3):255–75. doi: 10.1093/humupd/dmp050 19966268 PMC2849703

[10] Guelinckx I, Devlieger R, Beckers K, Vansant G. Maternal obesity: pregnancy complications, gestational weight gain and nutrition. Obesity reviews: an official journal of the International Association for the Study of Obesity. 2008;9(2):140–50. doi: 10.1111/j.1467-789X.2007.00464.x 18221480

[11] Gunderson EP, Abrams B. Epidemiology of gestational weight gain and body weight changes after pregnancy. Epidemiologic reviews. 1999;21(2):261–75. doi: 10.1093/oxfordjournals.epirev.a018001 10682262

[12] Voerman E, Santos S, Inskip H, Amiano P, Barros H, Charles MA, et al. Association of Gestational Weight Gain With Adverse Maternal and Infant Outcomes. Jama. 2019;321(17):1702–15. doi: 10.1001/jama.2019.3820 31063572 PMC6506886

[13] Goldstein RF, Abell SK, Ranasinha S, Misso M, Boyle JA, Black MH, et al. Association of Gestational Weight Gain With Maternal and Infant Outcomes: A Systematic Review and Meta-analysis. Jama. 2017;317(21):2207–25. doi: 10.1001/jama.2017.3635 28586887 PMC5815056

[14] Sunsaneevithayakul P, Titapant V, Ruangvutilert P, Sutantawibul A, Phatihattakorn C, Wataganara T, et al. Relation between gestational weight gain and pregnancy outcomes. The journal of obstetrics and gynaecology research. 2014;40(4):995–1001. doi: 10.1111/jog.12293 24428819

[15] Li N, Liu E, Guo J, Pan L, Li B, Wang P, et al. Maternal prepregnancy body mass index and gestational weight gain on pregnancy outcomes. PloS one. 2013;8(12):e82310. doi: 10.1371/journal.pone.0082310 24376527 PMC3869661

[16] Campos CAS, Malta MB, Neves PAR, Lourenço BH, Castro MC, Cardoso MA. Gestational weight gain, nutritional status and blood pressure in pregnant women. Revista de saude publica. 2019;53:57. doi: 10.11606/S1518-8787.2019053000880 31340349 PMC6629291

[17] Eraslan Sahin M, Col Madendag I. Effect of Gestational Weight Gain on Perinatal Outcomes in Low Risk Pregnancies with Normal Prepregnancy Body Mass Index. Biomed Res Int. 2019;2019:3768601. doi: 10.1155/2019/3768601 31355258 PMC6636447

[18] Fouelifack FY, Fouedjio JH, Fouogue JT, Sando Z, Fouelifa LD, Mbu RE. Associations of body mass index and gestational weight gain with term pregnancy outcomes in urban Cameroon: a retrospective cohort study in a tertiary hospital. BMC research notes. 2015;8:806. doi: 10.1186/s13104-015-1765-9 26686851 PMC4684922

[19] Asefa F, Cummins A, Dessie Y, Hayen A, Foureur M. Gestational weight gain and its effect on birth outcomes in sub-Saharan Africa: Systematic review and meta-analysis. PloS one. 2020;15(4):e0231889. doi: 10.1371/journal.pone.0231889 32324783 PMC7179909

[20] Esimai OA, Ojofeitimi E. Pattern and determinants of gestational weight gain an important predictor of infant birth weight in a developing country. Global journal of health science. 2014;6(4):148–54. doi: 10.5539/gjhs.v6n4p148 24999149 PMC4825478

[21] WHO. Trends in maternal mortality 2000 to 2017: estimates by WHO, UNICEF, UNFPA, World Bank Group and the United Nations Population Division. 2019.

[22] Kandala N-B, Stranges S. Geographic Variation of Overweight and Obesity among Women in Nigeria: A Case for Nutritional Transition in Sub-Saharan Africa. PloS one. 2014;9(6):e101103. doi: 10.1371/journal.pone.0101103 24979753 PMC4076212

[23] Liu L, Hong Z, Zhang L. Associations of prepregnancy body mass index and gestational weight gain with pregnancy outcomes in nulliparous women delivering single live babies. Scientific Reports. 2015;5(1):12863. doi: 10.1038/srep12863 26242798 PMC4525357

[24] Shao Y, Qiu J, Huang H, Mao B, Dai W, He X, et al. Pre-pregnancy BMI, gestational weight gain and risk of preeclampsia: a birth cohort study in Lanzhou, China. BMC pregnancy and childbirth. 2017;17(1):400. doi: 10.1186/s12884-017-1567-2 29191156 PMC5709979

[25] Ren M, Li H, Cai W, Niu X, Ji W, Zhang Z, et al. Excessive gestational weight gain in accordance with the IOM criteria and the risk of hypertensive disorders of pregnancy: a meta-analysis. BMC pregnancy and childbirth. 2018;18(1):281. doi: 10.1186/s12884-018-1922-y 29973178 PMC6030787

[26] Iyoke CA, Ugwu GO, Ezugwu FO, Lawani OL, Onyebuchi AK. Retrospective cohort study of the effects of obesity in early pregnancy on maternal weight gain and obstetric outcomes in an obstetric population in Africa. International journal of women’s health. 2013;5:501–7. doi: 10.2147/IJWH.S49909 23983492 PMC3747850

[27] Nwaogwugwu A, Simeon NA, Ekwuluo CE. Effect of excessive gestational weight gain on pregnancy outcome: A case study of tertiary hospitals in Benue State, Nigeria. International Journal of Research -GRANTHAALAYAH. 2020;8(3):180–9.

[28] ACOG. Weight Gain During Pregnancy. Committee on Obstetric Practice. 2013;548.

[29] Senbanjo OC, Akinlusi FM, Ottun TA. Early pregnancy body mass index, gestational weight gain and perinatal outcome in an obstetric population in Lagos, Nigeria. Pan Afr Med J. 2021;39:136–. doi: 10.11604/pamj.2021.39.136.25926 34527152 PMC8418156

[30] Nwaogwugwu A, Simeon NA, Ekwuluo CE. Effect of excessive gestational weight gain on pregnancy outcome: a case study of tertiary hospitals in Benue state, Nigeria. International Journal of Research -GRANTHAALAYAH. 2020;8(3):180–9.

[31] Olagbuji BN, Olofinbiyi BA, Akintayo AA, Aduloju OP, Ade-Ojo PI. Maternal perspectives on gestational weight gain: Critical information on developing weight control interventions. Nigerian medical journal: journal of the Nigeria Medical Association. 2015;56(2):96–102. doi: 10.4103/0300-1652.150689 25838623 PMC4382612

[32] Onwuka C, Ugwu E, Onah H, Obi S, Onwuka C, Menuba I, et al. Patterns of gestational weight gain and its association with birthweight in Nigeria. Nigerian journal of clinical practice. 2017;20(6):754–60. doi: 10.4103/1119-3077.208958 28656932

[33] Adeoye I.A., Bamgboye E.A., Omigbodun A.O. The Ibadan Pregnancy Cohort Study (IbPCS), a Prospective Cohort Study Protocol. African journal of biomedical research. 2022;25(2):273–80.

[34] WHO. Overweight and Obesity. World Health Organization. 2017.

[35] Fishman E, Böcker L, Helbich M. Adult Active Transport in the Netherlands: An Analysis of Its Contribution to Physical Activity Requirements. PloS one. 2015;10(4):e0121871. doi: 10.1371/journal.pone.0121871 25849902 PMC4388541

[36] Wanner M, Götschi T, Martin-Diener E, Kahlmeier S, Martin BW. Active transport, physical activity, and body weight in adults: a systematic review. American journal of preventive medicine. 2012;42(5):493–502. doi: 10.1016/j.amepre.2012.01.030 22516490

[37] WHO. Physical activity. World Health Organization. 2016.

[38] Chasan-Taber L, Schmidt MD, Roberts DE, Hosmer D, Markenson G, Freedson PS. Development and validation of a Pregnancy Physical Activity Questionnaire. Medicine and science in sports and exercise. 2004;36(10):1750–60. doi: 10.1249/01.mss.0000142303.49306.0d 15595297

[39] Chasan-Taber L, Silveira M, Pekow P, Braun B, Manson JE, Solomon CG, et al. Physical activity, sedentary behavior and risk of hypertensive disorders of pregnancy in Hispanic women. Hypertension in pregnancy. 2015;34(1):1–16. doi: 10.3109/10641955.2014.946616 25121645 PMC4810005

[40] Cox JL, Holden JM, Sagovsky R. Detection of postnatal depression: Development of the 10-item Edinburgh Postnatal Depression Scale. British Journal of Psychiatry. 1987;150:782–9. 10.1192/bjp.150.6.782 3651732

[41] Basu JK, Jeketera CM, Basu D. Obesity and its outcomes among pregnant South African women. International journal of gynaecology and obstetrics: the official organ of the International Federation of Gynaecology and Obstetrics. 2010;110(2):101–4. doi: 10.1016/j.ijgo.2010.02.020 20417513

[42] Sebire NJ, Jolly M, Harris JP, Wadsworth J, Joffe M, Beard RW, et al. Maternal obesity and pregnancy outcome: a study of 287,213 pregnancies in London. International journal of obesity and related metabolic disorders: journal of the International Association for the Study of Obesity. 2001;25(8):1175–82. 10.1038/sj.ijo.0801670 11477502

[43] Cnattingius S, Bergström R, Lipworth L, Kramer MS. Prepregnancy weight and the risk of adverse pregnancy outcomes. The New England journal of medicine. 1998;338(3):147–52. doi: 10.1056/NEJM199801153380302 9428815

[44] Ezeanochie MC, Ande AB, Olagbuji BN. Maternal obesity in early pregnancy and subsequent pregnancy outcome in a Nigerian population. African journal of reproductive health. 2011;15(4):55–9. 22571106

[45] Drehmer M, Duncan BB, Kac G, Schmidt MI. Association of second and third trimester weight gain in pregnancy with maternal and fetal outcomes. PloS one. 2013;8(1):e54704. doi: 10.1371/journal.pone.0054704 23382944 PMC3559868

[46] Hill B, Skouteris H, McCabe M, Fuller-Tyszkiewicz M. Body image and gestational weight gain: a prospective study. Journal of midwifery & women’s health. 2013;58(2):189–94. doi: 10.1111/j.1542-2011.2012.00227.x 23458678

[47] Jarman M, Yuan Y, Pakseresht M, Shi Q, Robson PJ, Bell RC. Patterns and trajectories of gestational weight gain: a prospective cohort study. CMAJ open. 2016;4(2):E338–45. doi: 10.9778/cmajo.20150132 27525254 PMC4966992

[48] Deputy NP, Andrea JS, Kim SY. Gestational Weight Gain—United States, 2012 and 2013. Morbidity and Mortality Weekly Report (MMWR). 2015;64(43):1215–20. doi: 10.15585/mmwr.mm6443a3 26540367 PMC4862652

[49] Silva TPRd Viana TGF, Pessoa MC Felisbino-Mendes MS, Inácio MLC Mendes LL, Velasquez-Melendez G Martins EF, Matozinhos FP. Environmental and individual factors associated with gestational weight gain. BMC public health. 2022;22(1):540. 35303846 10.1186/s12889-022-12948-w PMC8933974

[50] Angali KA, Shahri P, Borazjani F. Maternal dietary pattern in early pregnancy is associated with gestational weight gain and hyperglycemia: A cohort study in South West of Iran. Diabetes & Metabolic Syndrome: Clinical Research & Reviews. 2020;14(6):1711–7. 10.1016/j.dsx.2020.08.008 32916554

[51] Gillman MW. Interrupting Intergenerational Cycles of Maternal Obesity. Nestle Nutrition Institute workshop series. 2016;85:59–69. doi: 10.1159/000439487 27088333 PMC4913540

[52] Poston L. Maternal obesity, gestational weight gain and diet as determinants of offspring long term health. Best practice & research Clinical endocrinology & metabolism. 2012;26(5):627–39. doi: 10.1016/j.beem.2012.03.010 22980045

[53] Onwuka C, Ugwu E, Onah H, Obi S, Menuba I, Okafor I. Patterns of gestational weight gain and its association with birthweight in Nigeria. Nigerian journal of clinical practice. 2017;20(6):754–60. doi: 10.4103/1119-3077.208958 28656932

[54] Senbanjo OC, Akinlusi FM, Ottun TA. Early pregnancy body mass index, gestational weight gain and perinatal outcome in an obstetric population in Lagos, Nigeria. Pan Afr Med J. 2021;39:136. doi: 10.11604/pamj.2021.39.136.25926 34527152 PMC8418156

[55] Lartey A. Maternal and child nutrition in Sub-Saharan Africa: challenges and interventions. The Proceedings of the Nutrition Society. 2008;67(1):105–8. doi: 10.1017/S0029665108006083 18234138

[56] Sobal J, Stunkard AJ. Socioeconomic status and obesity: a review of the literature. Psychological bulletin. 1989;105(2):260–75. doi: 10.1037/0033-2909.105.2.260 2648443

[57] McLaren L. Socioeconomic status and obesity. Epidemiologic reviews. 2007;29:29–48. doi: 10.1093/epirev/mxm001 17478442

[58] Dinsa GD, Goryakin Y, Fumagalli E, Suhrcke M. Obesity and socioeconomic status in developing countries: a systematic review. Obesity reviews: an official journal of the International Association for the Study of Obesity. 2012;13(11):1067–79. doi: 10.1111/j.1467-789X.2012.01017.x 22764734 PMC3798095

[59] Paul KH, Graham ML, Olson CM. The web of risk factors for excessive gestational weight gain in low income women. Maternal and child health journal. 2013;17(2):344–51. doi: 10.1007/s10995-012-0979-x 22415812 PMC4535446

[60] WHO. Depression fact sheet. 2021.

[61] Jarde A, Morais M, Kingston D, Giallo R, MacQueen GM, Giglia L, Beyene J, Wang Y, McDonald SD. Neonatal Outcomes in Women With Untreated Antenatal Depression Compared With Women Without Depression: A Systematic Review and Meta-analysis. JAMA psychiatry. 2016;73(8):826–37. doi: 10.1001/jamapsychiatry.2016.0934 27276520

[62] Adeniyi AF, Ogwumike OO. Physical activity and energy expenditure: findings from the Ibadan Pregnant Women’s Survey. African journal of reproductive health. 2014;18(2):117–26. 25022148

[63] Adeoye IA. Pattern and correlates of physical activity and sedentary behaviours of pregnant women in Ibadan, Nigeria: Findings from Ibadan pregnancy cohort study. PLOS Global Public Health. 2022;2(10):e0001153. doi: 10.1371/journal.pgph.0001153 36962637 PMC10021993

[64] Baker JH, Rothenberger SD, Kline CE, Okun ML. Exercise during early pregnancy is associated with greater sleep continuity. Behavioral sleep medicine. 2018;16(5):482–93. doi: 10.1080/15402002.2016.1228649 27739877 PMC6124311

[65] da Silva SG, Ricardo LI, Evenson KR, Hallal PC. Leisure-time physical activity in pregnancy and maternal-child health: a systematic review and meta-analysis of randomized controlled trials and cohort studies. Sports medicine. 2017;47(2):295–317. doi: 10.1007/s40279-016-0565-2 27282925

[66] Russo LM, Nobles C, Ertel KA, Chasan-Taber L, Whitcomb BW. Physical activity interventions in pregnancy and risk of gestational diabetes mellitus: a systematic review and meta-analysis. Obstetrics & Gynecology. 2015;125(3):576–82. doi: 10.1097/AOG.0000000000000691 25730218

[67] Papatriantafyllou E, Efthymiou D, Zoumbaneas E, Popescu CA, Vassilopoulou E. Sleep Deprivation: Effects on Weight Loss and Weight Loss Maintenance. Nutrients. 2022;14(8). doi: 10.3390/nu14081549 35458110 PMC9031614

[68] Malik VS, Schulze MB, Hu FB. Intake of sugar-sweetened beverages and weight gain: a systematic review. The American journal of clinical nutrition. 2006;84(2):274–88. doi: 10.1093/ajcn/84.1.274 16895873 PMC3210834

[69] Luger M, Lafontan M, Bes-Rastrollo M, Winzer E, Yumuk V, Farpour-Lambert N. Sugar-Sweetened Beverages and Weight Gain in Children and Adults: A Systematic Review from 2013 to 2015 and a Comparison with Previous Studies. Obesity facts. 2017;10(6):674–93. doi: 10.1159/000484566 29237159 PMC5836186

[70] Maslova E, Halldorsson TI, Astrup A, Olsen SF. Dietary protein-to-carbohydrate ratio and added sugar as determinants of excessive gestational weight gain: a prospective cohort study. BMJ open. 2015;5(2):e005839. doi: 10.1136/bmjopen-2014-005839 25670731 PMC4325128

[71] Malik VS, Hu FB. The role of sugar-sweetened beverages in the global epidemics of obesity and chronic diseases. Nature Reviews Endocrinology. 2022;18(4):205–18. doi: 10.1038/s41574-021-00627-6 35064240 PMC8778490

[72] Nohr EA, Vaeth M, Baker JL, Sørensen T, Olsen J, Rasmussen KM. Combined associations of prepregnancy body mass index and gestational weight gain with the outcome of pregnancy. The American journal of clinical nutrition. 2008;87(6):1750–9. doi: 10.1093/ajcn/87.6.1750 18541565

[73] Cedergren M. Effects of gestational weight gain and body mass index on obstetric outcome in Sweden. International journal of gynaecology and obstetrics: the official organ of the International Federation of Gynaecology and Obstetrics. 2006;93(3):269–74. doi: 10.1016/j.ijgo.2006.03.002 16626716

[74] Langford A, Joshu C, Chang JJ, Myles T, Leet T. Does Gestational Weight Gain Affect the Risk of Adverse Maternal and Infant Outcomes in Overweight Women? Maternal and child health journal. 2011;15(7):860–5. doi: 10.1007/s10995-008-0318-4 18247109

[75] Gaillard R, Durmuş B, Hofman A, Mackenbach JP, Steegers EA, Jaddoe VW. Risk factors and outcomes of maternal obesity and excessive weight gain during pregnancy. Obesity (Silver Spring, Md). 2013;21(5):1046–55. doi: 10.1002/oby.20088 23784909

[76] Poston L, Bell R, Croker H, Flynn AC, Godfrey KM, Goff L, et al. Effect of a behavioural intervention in obese pregnant women (the UPBEAT study): a multicentre, randomised controlled trial. The lancet Diabetes & endocrinology. 2015;3(10):767–77. doi: 10.1016/S2213-8587(15)00227-2 26165396

[77] Bhattacharya S, Campbell DM, Liston WA, Bhattacharya S. Effect of Body Mass Index on pregnancy outcomes in nulliparous women delivering singleton babies. BMC public health. 2007;7(1):168. doi: 10.1186/1471-2458-7-168 17650297 PMC1940246

[78] Fyfe EM, Thompson JMD, Anderson NH, Groom KM, McCowan LM. Maternal obesity and postpartum haemorrhage after vaginal and caesarean delivery among nulliparous women at term: a retrospective cohort study. BMC pregnancy and childbirth. 2012;12(1):112. doi: 10.1186/1471-2393-12-112 23078042 PMC3495044

[79] Butwick AJ, Abreo A, Bateman BT, Lee HC, El-Sayed YY, Stephansson O, Flood P. Effect of Maternal Body Mass Index on Postpartum Hemorrhage. Anesthesiology. 2018;128(4):774–83. doi: 10.1097/ALN.0000000000002082 29346134 PMC5849500

[80] Blomberg M. Maternal Obesity and Risk of Postpartum Hemorrhage. Obstetrics & Gynecology. 2011;118(3):561–8. doi: 10.1097/AOG.0b013e31822a6c59 21860284

