# Supplementary material for: Gestational weight gain among pregnant women in Ibadan, Nigeria: Pattern, predictors and pregnancy outcomes
Source: PLoS One. 2023 Aug 18;18(8):e0290102. doi: 10.1371/journal.pone.0290102 (PMC10437817; doi:10.1371/journal.pone.0290102)
Supplement: Attachment — Submitted filename: R1_POINT BY POINT RESPONSE_GWG plosone.docx [file pone.0290102.s002.docx]

POINT-BY-POINT RESPONSE TO THE REVIEWER'S COMMENT

**"Gestational Weight Gain among Pregnant women in Ibadan, Nigeria: pattern, predictors and pregnancy outcomes"**

Reviewer #1:

This study determines factors affecting gestational weight gain and its association with maternal outcomes in Nigeria. Please find below my comments.

Abstract

The results did not reflect all findings from the study, e.g., findings from Table 4

- Table 4 has been removed from the manuscript

Introduction

- Overall, the introduction section is slightly general without the support of facts or evidence, such as percentages or rates. This information is important to support the study's rationale and for the reader to understand the actual situation of problems. Suggest including some background information on the GWG pattern in Nigeria and other related regions
- The introduction is re-written and supported with evidence

a. Line 124-129: Suggest elaborate these statements, e.g., What is the percentage of excessive and insufficient in the countries?

- Done

b. Line 129-131: please elaborate

- Done

Figure: Suggest replacing Figure 1 with the flow chart of study participants, whereby how the study participants were selected.

- Figure 1 is now a flow chart of study participants

Materials and Methods:

- Line 164: …at investing.., suggest …to investigate.
- Replaced with "to investigate."
- Line 215- 254: Since lifestyle information was collected in this study, especially the sugar-sweetened beverages, and sleep pattern, and physical activity (line 178-180), wondering why the authors did not investigate the associations between lifestyle factors and GWG.
- Additional lifestyle factors added and presented in Tables 1 & 2
  - sugar-sweetened beverage intake
  - sleep pattern
  - physical activity

*Importantly, our study investigated the influence of maternal lifestyle characteristics – SSB intake, sleep pattern and physical on GWG among our study population. We found that less physically active women had higher odds for excessive GWG than more physically active women. The WHO recommends that pregnant women engage in at least 150 minutes of moderate-intensity physical activity during the week [50]. Researchers have reported poor compliance with the recommendation among Nigerian pregnant women [51, 52]. Hence the need to actively promote physical activity among pregnant women to obtain benefits, including improving cardiovascular fitness, preventing excessive GWG and GDM, improving sleep quality and so on [53-55]. Although there was a significant relationship between sleep duration and GWG, women with excessive GWG had a shorter sleep duration than those with adequate and insufficient sleep. Researchers have found an association between sleep deprivation and weight gain. Further studies should further explore this relationship among the Nigerian pregnant population.*

How the variables were selected to be included in the model

- *Variables significant at a 5% level of statistical significance at binary logistic analysis were subjected to multiple logistic regression analyses (religion, income, BMI, physical activity level, and APD). The estimated unadjusted and adjusted odds ratios, 95% confidence intervals and p-values (p<0.05) of associated factors were reported.*

Line 256: please list out the adjusted variables

- Done

Line 209: please include the source of reference

- Done

Results:

1. Line 315: "'20,000-99,999', please provide a footnote on the rate of USD for ease of interpretation

- a footnote on the rate of USD provided in Table 1

Discussion:

Line 406: had appropriate weight.., suggest normal GWG

- Replaced with Normal GWG

Line 423-430: Since the authors noticed that maternal lifestyle behaviours, such as nutrition and physical activity, are important factors contributing to GWG, suggesting the author included these factors in the analysis (Table 2)

- Added to the analysis

Line 451- 455: please include the source of reference; what is the recommended GWG practice in Nigeria? Suggest including this information in the introduction 10.

Line 472-474: Any information on the types of cesarean sections performed? Elective or emergency cesarean section

- The direction and association with GWG was similar for All cesarean sections, Elective or emergency cesarean section

Reviewer #2

I would like to thank the authors for conducting the study on this important and timely topic. Please find my specific comments below.

1. Better to clearly report what type of logistic regression (binary or multinomial) was conducted to examine associations.

- Binary logistic regression performed and reported in the manuscript

2. The authors defined abbreviations multiple times. For example, "Gestational weight gain (GWG)" was defined many times. Please revisit the use of abbreviations.

- use of abbreviations revisited Gestational weight gain (GWG) now defined once

3. The author bluntly reported the last weight as "the third trimester weight", which is vague. They need to report the exact gestational week at which the last maternal weight measured.

- .According to the literature there are three ways of estimating GWG
  - *Total weight gain – This is the difference the woman’s weight just before the onset of labour and her pre-pregnancy weight. (These pieces of information are not readily available in settings with poor medical record keeping, lack of awareness of health workers on the importance of GWG, high out of hospital delivery. This situation is prevalent in several hospitals.*
  - *Net weight gain – This is the difference the woman’s total weight gain and birthweight of the infant*
  - *Rate of weight gain per week – This is a measure weight gain over a particular time period in weeks divided by the time interval in weeks. This rate could be assessed within the 2nd and third trimesters. This measure would appeal to researchers from developing countries, which was the measure used in this study, where GWG rate as assessed as the difference between the final weight recorded by the woman during antenatal care in the third trimester and the weight at the booking visit.*
- In this study we used the rate of weight gain per week because weight at delivery was not assessible
- That is the final third-trimester weight minus booking weight divided by gestational age, at which the final third-trimester weight was measured minus gestational age at booking.

GWG rate = $\frac{third trimester weight \left( final \right)- weight at booking}{GA at third trimester weight \left( final \right)- GA at booking}$

4. They also stated that "The factors associated with insufficient and excessive GWG were assessed using bivariate logistic regression" What do they mean by "bivariate logistic regression?"

- binary logistic regression

4. In table 3, the authors chose insufficient GWG as reference group while examining the association between GWG and pregnancy outcomes. Since insufficient and excessive GWG are undesirable outcomes, they are supposed to compare their outcomes with the outcomes of adequate GWG. I would suggest them to change the reference group from insufficient weight gain to adequate GWG and report the effect of insufficient and excessive GWG on pregnancy outcomes.

- Adequate GWG now the reference group

5. I would also suggest them to report whether the finding in the table three are crude or adjusted. If they have adjusted for confounders, they need to report the list of variables for which the analysis were adjusted.

- These are unadjusted estimates – indicated

6. Similarly, the findings in table 4 were a bit confusing. No reference group. It is unclear what "-" mean.
   - Table 4 now removed

6. It would have been good if the authors reported how many of the participants were underweight, normal weight, overweight and obese.

- Now reported.
- Underweight (36);; Normal weight (622), overweight (337), obese (232)

7. It would be good if the authors commented on the implication and appropriateness of using the IOM recommendations for GWG for Nigerian women.

- Comment added
  - *“The IOM guidelines are not yet in place, nor is there any consensus on GWG in Nigeria's maternal care [24, 28]. For instance, based on the IOM guidelines, the American College of Obstetricians and Gynaecologists recommends that clinicians calculate a woman's pre-pregnancy BMI at the first antenatal care visit and educate her on the importance of appropriate GWG goals [25]. Adopting and implementing a GWG guideline in Nigeria's maternal health services is essential. Although the IOM guideline is based on cut-off points from white women and is yet to be validated among Africans, the IOM guidelines are still the most utilised GWG guideline. Hence it could be helpful in Nigeria up until local guidelines are developed”*

7. Gestational weight gain was measured as the difference between the last third trimester weight recorded and the booking weight divided by the number of weeks between the weights. The authors need to substantiate the appropriateness of this GWG calculation method with evidence.
8. The gestational age for booking weight was 20 weeks. Does this time have any implication of GWG?
