# Supplementary material for: Gestational weight gain among pregnant women in Ibadan, Nigeria: Pattern, predictors and pregnancy outcomes
Source: PLoS One. 2023 Aug 18;18(8):e0290102. doi: 10.1371/journal.pone.0290102 (PMC10437817; doi:10.1371/journal.pone.0290102)
Supplement: Attachment — Submitted filename: Reviewer comments2.docx [file pone.0290102.s003.docx]

Please find below my comments:

1. Line 154: …..”
2. Line 154-155: The statement is not clear or scientifically precise. Suggesting “Furthermore, the current body of evidence on gestational weight gain (GWG) in Nigeria is limited, with only a few emerging studies available [29-32].”
3. Please include the write-up or description of Figure 1 in the methodology section, as it is important to mention that 1254 out of 1745 participants were included in the final analysis.
4. Line 199-200: Suggest rephrasing this statement to improve consistency in terms of presenting the time intervals and stages of follow-up for the study participants
5. Line 201-202: suggest including the mean and standard deviation for the week of gestation during the third trimester
6. Line 216: “gestational weight gain” Please ensure consistency in the usage of terms throughout the entire write-u
7. Line 218-220: It is recommended to rephrase the statement to align with scientific writing principles
8. Line 222-225: Suggest rephrasing these statements. It is important to mention that the Institute of Medicine (IOM) guidelines provide recommendations for weight gain during pregnancy based on a woman's pre-pregnancy body mass index (BMI). Consequently, it is relevant to connect this information to the subsequent statement
9. Line 248-249: please provide a reference for the birth weight categories. Same comments for gestational age at delivery, APGAR and Postpartum Haemorrhage
10. Line 272-274: Please include the “Forest plots showing the association of specific pregnancy outcomes with gestational weight gain (GWG) based on maternal BMI” in the statistical analysis section. Additionally, clarify the reason why the underweight group is not included in this analysis.
11. Line 288: Does the term "physical activity" refer to motorized transport in the table? If it does, please ensure consistent use of terms to avoid confusion.
12. Line 289-291: The statement, "Specifically, women with higher education and income experienced more excessive gestational weight gain (GWG) than insufficient or adequate GWG compared to women with lower education and income," is incorrect. It is important to note that chi-square tests only determine the overall association and do not specifically examine associations within specific categories. Therefore, this statement should be revised to avoid implying specific associations within subcategories based on chi-square analysis.
13. Line 295-296: The information mentioned cannot be found in the table.
14. Table 1:
    a. “Prevalence” The term "prevalence" may not be suitable in this context. I suggest using the term "proportion" instead. "Prevalence" typically refers to the occurrence of a disease or illness, whereas adequate weight gain is not a disease but rather a desirable outcome. Therefore, using "proportion" would be more appropriate to describe the occurrence or proportion of participants with adequate weight gain.
    b. Please check again the grouping for age (> 35 years), income or household income, motorized transport (yes/no or physically active/physically inactive)
    c. Please rearrange the sequence of the variables, starting with socio-demographic variables and followed by lifestyle characteristics. Additionally, please ensure that the group categorizations are consistent with the write-up in lines 230-237. Ethnicity is not mentioned in the text.
    d. Since new variables have been included in Table 1, it is suggested to add the corresponding write-up in the text
15. Figure 2: Please review the percentages again, as the total percentage for normal weight and overweight appears to exceed 100%
16. Line 352-355: It is suggested to also include the finding on excessive GWG.
17. Line 353-354: Suggest rephrasing to avoid starting the sentence with "and"
18. Line 375-383: Suggest adding a statement to mention that no significant associations were observed between pregnancy outcomes and gestational weight gain. This helps prevent potential misinterpretation or assumptions of associations that do not exist, ensuring the accuracy and reliability of the study's conclusions.
19. Line 386: “maternal body mass index”. Please use the abbreviation "BMI" for maternal body mass index, as it is important to maintain consistency throughout the text.
20. Figure 3:
    a. Please ensure consistency by using the same term, such as “spontaneous vertex or spontaneous vaginal delivery” throughout the entire text. This will help avoid confusion and maintain clarity in the discussion.
21. Line 427-429: Please include reference for this statement
22. Line 435: 29.% formatting error
23. Line 450: "correct GWG," it would be better to use "interventions encompass creating awareness of the appropriate gestational weight gain (GWG) range."
24. Line 455: "Gestational weight gain" itself is not a problem, but it can be a risk factor for adverse outcomes if it falls into the category of inappropriate or excessive weight gain. Therefore, it would be more accurate to refer to the interventions as targeting the risk factors for inappropriate gestational weight gain rather than gestational weight gain itself. This clarification highlights the importance of addressing the factors that contribute to undesirable weight gain during pregnancy. risk factor for inappropriate GWG Please revise accordingly
25. Line 503: There is no discussion on SSB
26. Line 562-563: rephrase the statement as this study did not find any significant association between excessive GWG and outcomes
27. Line 504-511: The variable of physical activity in this study pertains specifically to motorized transport. However, it is essential to note that physical activity encompasses more than just transportation-related activities. Therefore, in the methodology section, it is necessary to provide a clear definition of physical activity that encompasses a broader range of activities. Additionally, it is important to describe how this motorized transport variable is linked to the subsequent statement. Providing references that support the chosen definition and the association between motorized transport and physical activity would further enhance the clarity and validity of the study.
28. Line 529-530: Please include the reference for this statement
29. Line 537-538: Please include a discussion on this
30. Line 539-540: Please revise the statement to remove the dash
31. Line 547: suggest rephrasing the statement as the inclusion of alcohol consumption as a psychological factor is incorrect
