# Supplementary material for: Gestational weight gain among pregnant women in Ibadan, Nigeria: Pattern, predictors and pregnancy outcomes
Source: PLoS One. 2023 Aug 18;18(8):e0290102. doi: 10.1371/journal.pone.0290102 (PMC10437817; doi:10.1371/journal.pone.0290102)
Supplement: Attachment — Submitted filename: Point by point response to R comments R2.docx [file pone.0290102.s004.docx]

**POINT BY POINT RESPONSE TO THE REVIEWERS COMMENTS**

Reviewer 1

1. Line 154: …..”
   - Done
2. Line 154-155: The statement is not clear or scientifically precise. Suggesting “Furthermore, the current body of evidence on gestational weight gain (GWG) in Nigeria is limited, with only a few emerging studies available [29-32].”
   - Done
3. Please include the write-up or description of Figure 1 in the methodology section, as it is important to mention that 1254 out of 1745 participants were included in the final analysis.
   - description of Figure 1 included
     - *The flowchart of study participants from enrolment to delivery is shown in fig 1 and 1254 out of the 1745 participants were included in the final analysis.*
4. Line 199-200: Suggest rephrasing this statement to improve consistency in terms of presenting the time intervals and stages of follow-up for the study participants
   - Rephrased
     - *After recruitment (≤ 20 weeks), study participants were followed up at three time points: 24 -28 weeks, third trimester, at delivery.*
5. Line 201-202: suggest including the mean and standard deviation for the week of gestation during the third trimester
   - the mean and standard deviation for the week of gestation during the third trimester included
     - *Serial weight values were used to estimate GWG according to the Institute of Medicine guidelines (2009) [1] which was the difference between the booking weight and the last weight assessed during the third trimester at the ANC (32.4 ± 4.7 weeks) .*
6. Line 216: “gestational weight gain” Please ensure consistency in the usage of terms throughout the entire write-up
   - Effected – GWG used throughout the manuscript after the first mention of “gestational weight gain”
7. Line 218-220: It is recommended to rephrase the statement to align with scientific writing principles
   - Rephrased

*The GWG rate was calculated as the difference between the last third-trimester weight recorded and the booking weight divided by the number of gestational weeks between in the interval these weights were measured as annotated as follows.*

- *GWG rate =* $\frac{third trimester weight \left( final \right)- weight at booking}{GA at third trimester weight \left( final \right)- GA at booking}$

8. Line 222-225: Suggest rephrasing these statements. It is important to mention that the Institute of Medicine (IOM) guidelines provide recommendations for weight gain during pregnancy based on a woman's pre-pregnancy body mass index (BMI). Consequently, it is relevant to connect this information to the subsequent statement
   - DONE, Sir
     - *The Institute of Medicine (IOM) guidelines provide recommendations for weight gain during pregnancy based on a woman's pre-pregnancy body mass index (BMI). Hence, we categorised the GWG rate into insufficient, adequate, and excessive weight gain.*
9. Line 248-249: please provide a reference for the birth weight categories. Same comments for gestational age at delivery, APGAR and Postpartum Haemorrhage
   - Provide references.
     - *Basu JK, Jeketera CM, Basu D. Obesity and its outcomes among pregnant South African women. International journal of gynaecology and obstetrics: the official organ of the International Federation of Gynaecology and Obstetrics. 2010;110(2):101-4.*
     - *Sebire NJ, Jolly M, Harris JP, Wadsworth J, Joffe M, Beard RW, Regan L, Robinson S. Maternal obesity and pregnancy outcome: a study of 287,213 pregnancies in London. International journal of obesity and related metabolic disorders : journal of the International Association for the Study of Obesity. 2001;25(8):1175-82.*
     - *Cnattingius S, Bergström R, Lipworth L, Kramer MS. Prepregnancy weight and the risk of adverse pregnancy outcomes. The New England journal of medicine. 1998;338(3):147-52.*
     - *Ezeanochie MC, Ande AB, Olagbuji BN. Maternal obesity in early pregnancy and subsequent pregnancy outcome in a Nigerian population. African journal of reproductive health. 2011;15(4):55-9.*
10. Line 272-274: Please include the “Forest plots showing the association of specific pregnancy outcomes with gestational weight gain (GWG) based on maternal BMI” in the statistical analysis section. Additionally, clarify the reason why the underweight group is not included in this analysis.
    - Added in the statistical analysis section as ….
      - *Forest plots showing the association of specific pregnancy outcomes with GWG based on maternal BMI” were also presented.*
    - why the underweight group
      - Footnote for the forest plots
      - *Underweight weight was absent from the forest plots because they made up a very low proportion of the final analysis 36 (2.9%) and was mostly omitted from the output of the stratified analysis for the forest plots.*
11. Line 288: Does the term "physical activity" refer to motorized transport in the table? If it does, please ensure consistent use of terms to avoid confusion.
    - Harmonized and defined
12. Line 289-291: The statement, "Specifically, women with higher education and income experienced more excessive gestational weight gain (GWG) than insufficient or adequate GWG compared to women with lower education and income," is incorrect. It is important to note that chi-square tests only determine the overall association and do not specifically examine associations within specific categories. Therefore, this statement should be revised to avoid implying specific associations within subcategories based on chi-square analysis.
    - Corrected and recast
    - *Specifically, the level of education and income had a positive association with excessive GWG i.e. women with primary education or less: 15 (45.5 %), secondary 151 (44.0 %), and tertiary 537 (61.4%) and income: < 20,000 - 192 (46.7%), "20,000 – 99,999", 373 (63.2%), and ≥ 100,000 48 (61.5%).*
13. Line 295-296: The information mentioned cannot be found in the table.
    - Highlighted in table 1
      - *Also, more Christians (60.7%) than Muslims (49.6 %) experienced excessive GWG. Women excessive GWG spent significantly less time on moderate intensity activity per week (25.3±23.1) minutes, more physically inactive (60.7%) and more sedentary ( 61.0) than women with inadequate GWG who spent (28.4 ± 23.6) minutes/week on moderate intensity activity, less more physically inactive (52.0%) and less sedentary (52.3%). Conversely, women who experienced antepartum depression (APD) had a higher proportion of insufficient GWG (39.2%) compared to women without APD (24.9%)*
14. Table 1:
i. . “Prevalence” The term "prevalence" may not be suitable in this context. I suggest using the term "proportion" instead. "Prevalence" typically refers to the occurrence of a disease or illness, whereas adequate weight gain is not a disease but rather a desirable outcome. Therefore, using "proportion" would be more appropriate to describe the occurrence or proportion of participants with adequate weight gain.

- Changed to PROPORTION

ii. Please check again the grouping for age (> 35 years), income or household income, motorized transport (yes/no or physically active/physically inactive)

- Grouping checked
  - < 35 years (not > 35 years)
  - Further details physical activity provided

iii. Please rearrange the sequence of the variables, starting with socio-demographic variables and followed by lifestyle characteristics. Additionally, please ensure that the group categorizations are consistent with the write-up in lines 230-237. Ethnicity is not mentioned in the text.
     - Since new variables have been included in Table 1, it is suggested to add the corresponding write-up in the text
     - Ethnicity is now mentioned in the text.
       - *The mean age of study participants was x ± SD and majority belonged to the Yoruba ethnic group (88.8%).*
15. Figure 2: Please review the percentages again, as the total percentage for normal weight and overweight appears to exceed 100%
    - Errors now corrected all percentages now add up to 100%
16. Line 352-355: It is suggested to also include the finding on excessive GWG.
    - Findings included
      - *Specifically, the odds of excessive GWG was associated with increasing revenue' 20,000-99,999' (AOR: 1.64, 95% CI: 1.25 – 2.17), '≥ 100,000' (AOR: 1.54, 95 CI: 0.90 – 2.64), compared to those who earned less than 20,000. Compared with women with normal weight, women with overweight (AOR: 2.07, 95% CI 1.52 – 2.88) and obese (AOR: 1.56, 95% CI 1.11– 2.20) had a higher odds of excessive weight gain. Also women who had depression during pregnancy were also less likely to experience excessive GWG (AOR: 0.56, 95% CI 0.36 – 0.82). Conversely, compared with Christian women, Muslims had higher odds for insufficient GWG (AOR: 1.52, 95% CI 1.13 – 2.05). And women with APD had 70% higher odds of insufficient GWG (AOR: 1.70, 95% CI 1.17 – 2.45) than women without APD.*
17. Line 353-354: Suggest rephrasing to avoid starting the sentence with "and"
    - “And” is removed
18. Line 375-383: Suggest adding a statement to mention that no significant associations were observed between pregnancy outcomes and gestational weight gain. This helps prevent potential misinterpretation or assumptions of associations that do not exist, ensuring the accuracy and reliability of the study's conclusions.
    - Added, thanks Sir
      - *There were no statistically significant associations observed between pregnancy outcomes and gestational weight gain.*
19. Line 386: “maternal body mass index”. Please use the abbreviation "BMI" for maternal body mass index, as it is important to maintain consistency throughout the text.
    - "BMI" for maternal body mass index in the text
20. Figure 3:

Please ensure consistency by using the same term, such as “spontaneous vertex or spontaneous vaginal delivery” throughout the entire text. This will help avoid confusion and maintain clarity in the discussion.

- “spontaneous vaginal delivery used throughout the manuscript”

21. Line 427-429: Please include reference for this statement
    - Canada, Australia and Brazil - DONE
22. Line 435: 29.% formatting error
    - DONE – 29.4%
23. Line 450: "correct GWG," it would be better to use "interventions encompass creating awareness of the appropriate gestational weight gain (GWG) range."
    - Change effected
      - *These interventions encompass creating awareness on the appropriate gestational weight gain (GWG) range,*
24. Line 455: "Gestational weight gain" itself is not a problem, but it can be a risk factor for adverse outcomes if it falls into the category of inappropriate or excessive weight gain. Therefore, it would be more accurate to refer to the interventions as targeting the risk factors for inappropriate gestational weight gain rather than gestational weight gain itself. This clarification highlights the importance of addressing the factors that contribute to undesirable weight gain during pregnancy. risk factor for inappropriate GWG Please revise accordingly
    - Revised accordingly
      - *Investigating the risk factors associated with inappropriate GWG is essential for providing targeted interventions for addressing the factors that contribute to undesirable weight gain during pregnancy among Nigerian women.*
25. Line 503: There is no discussion on SSB
    - Added
      - *In addition, although our study did not find any significant relationship between SSB intake and GWG, SSBs have been associated with obesity, weight gain and excessive GWG in the literature [2-4] because of the high and easily absorbed sugar content that leads to high energy intake and weight gain [5].*
26. Line 562-563: rephrase the statement as this study did not find any significant association between excessive GWG and outcomes
    - DONE
      - *Even though the incidences of caesarean section and postpartum haemorrhage were higher among women with excessive GWG compared with adequate and insufficient GWG, we did not find any significant association between excessive GWG and pregnancy outcomes.*
27. Line 504-511: The variable of physical activity in this study pertains specifically to motorized transport. However, it is essential to note that physical activity encompasses more than just transportation-related activities. Therefore, in the methodology section, it is necessary to provide a clear definition of physical activity that encompasses a broader range of activities. Additionally, it is important to describe how this motorized transport variable is linked to the subsequent statement. Providing references that support the chosen definition and the association between motorized transport and physical activity would further enhance the clarity and validity of the study.
    - ADDRESSED
      - *METHOD*
      - *The participants’ level of physical activity is assesses using the (i.) passive transport (ownership of motorized transport) i.e. physically inactive while active transport or walking as physically active)[6, 7] (ii.) Duration of moderate-intensity exercise (minutes per week) is the total time spent on moderate-intensity activity per week[8]. (iii.) Sedentary behaviour was also assessed using the pregnancy physical activity questionnaire (PPAQ) and classified as high or low according to the PPAQ instruction guide [9, 10].*

| **Physical Activity** |  |
| --- | --- |
| Physically active | 635(50.6) |
| Physically inactive | 619(49.4) |
| **Sedentary Behaviour** |  |
| Low | 561 (52.9) |
| High | 518 (48.1) |
| **Moderate intensity activity duration (minutes)** | 25.8±22.7 |

- *RESULT*
- *Women excessive GWG spent significantly less time on moderate intensity activity per week (25.3±23.1) minutes, more physically inactive (60.7%) and more sedentary ( 61.0%) than women with inadequate GWG who spent (28.4 ± 23.6) minutes/week on moderate intensity activity, less more physically inactive (52.0%) and less sedentary (52.3%).*
- *DISCUSSION*
- *We assessed physical activity using active/passive transport [6], duration of moderate-intensity exercise [6, 8] and sedentary behaviour [9, 10]. Active transport is a measure of physical activity because it contributes to total physical activity and increases energy expenditure [6, 7]. Women excessive GWG spent less time on moderate intensity activity per week minutes, more physically inactive and more sedentary than women with inadequate GWG. We found that physically in active and sedentary women had higher odds for excessive GWG than physically active women. Although, the relationship became insignificant on multivariate analysis. Importantly, the WHO recommends that pregnant women engage in at least 150 minutes of moderate-intensity physical activity during the week [8]. Researchers have reported poor compliance with the recommendation among Nigerian pregnant women [11, 12]. Hence the need to actively promote physical activity among pregnant women to obtain benefits, including improving cardiovascular fitness, preventing excessive GWG and GDM, improving sleep quality and so on [13-15].*

28. Line 529-530: Please include the reference for this statement
    - DONE
29. Line 537-538: Please include a discussion on this

- Discussion on Obesity and Post-partum haemorrhage is included
  - *The association between maternal obesity postpartum haemorrhage has been reported in the literature [16-18]. The plausible reasons include poor uterine contractility in obese women compared with non-obese [19], increase foetal weight that could lead to uterine atony or perineal tear, associate large placenta and placenta praevia [18]. Blomberg et al. in the cohort study among Swedish pregnancy reported that the risk of atonic uterine haemorrhage increased rapidly with BMI [20]. Therefore, extra vigilance is advised in the active management of the third stage of labour in obese women with excessive GWG [18, 19].*

30. Line 539-540: Please revise the statement to remove the dash
    - DONE
      - *This study contributes significantly to a critical gap in maternal health literature in Nigeria, in which GWG has been scantly examined.*
31. Line 547: suggest rephrasing the statement as the inclusion of alcohol consumption as a psychological factor is incorrect
    - DONE
      - *We investigated the influence of a broader range of variables; sociodemographic, lifestyle (alcohol consumption, tobacco exposure, SSB intake, physical activity and sleep duration), healthcare utilisation and psychological factors such as APD, which were lacking in previous studies.*

Reviewer #2:

I would like to express my gratitude to the authors for revising the paper and resubmitting it. However, I have noticed that the authors only addressed certain parts of my comments.

- **Thank you for your support for our publication we apologize for the omission which was a complete oversight**

1. One specific example is my suggestion to substantiate their gestational weight gain calculation methods with evidence. I had recommended that they discuss the appropriateness of the chosen method in their specific context, its limitations, and support their claims by citing existing evidence. Although the authors attempted to address this in the response section, they failed to provide substantial evidence to support their claims. Furthermore, they neglected to discuss this matter in the manuscript itself.
   - Issues are addressed as follows

- *In this study, we assessed GWG using the rate of weight gain per week rather than the total weight gain, even though a more precise measure, because information required for the estimation total weight gain i.e. the weight just before the onset of labour and her pre-pregnancy weight, were not available in our study setting. Hence, in such a situation IOM guidelines recommends rate of weight gain per week assessed within the two and third trimester [1, 21, 22] which has also been put to use by other researchers in other settings [1, 21, 23, 24]*.
  - *Rasmussen KM, Yaktine ALE. Weight Gain During Pregnancy: Reexamining the Guidelines.Institute of Medicine. In: Rasmussen KM, Yaktine AL, editors. Weight Gain During Pregnancy: Reexamining the Guidelines. Washington (DC): National Academies Press (US) Copyright © 2009, National Academy of Sciences.; 2009.*
  - *Siega-Riz AM, Bodnar LM, Stotland NE, Stang. The Current Understanding of Gestational Weight Gain among Women with Obesity and the Need for Future Research. NAM Perspectives Discussion Paper,. National Academy of Medicine, Washington, DC. 201*
  - *Drehmer M, Duncan BB, Kac G, Schmidt MI. Association of second and third trimester weight gain in pregnancy with maternal and fetal outcomes. PloS one. 2013;8(1):e54704.*
  - *Campos CAS, Malta MB, Neves PAR, Lourenço BH, Castro MC, Cardoso MA. Gestational weight gain, nutritional status and blood pressure in pregnant women. Revista de saude publica. 2019;53:57.*

2. Another concern I raised was regarding the implications of using booking weight at 20 weeks of gestation. Unfortunately, the authors did not respond to this concern or address the issue in the manuscript. It is important to note that 20 weeks of gestation falls in the middle of the second trimester, during which women typically experience a significant amount of weight gain that could potentially impact the calculation of gestational weight gain in the manuscript. This issue should have been clearly discussed, at the very least, as a limitation of the study.

- Issues addressed in the limitation
  - *In this study, we could not estimate total GWG because prepregnancy and delivery weights were unavailable; hence, we used a weekly GWG rate. Also, the use of booking weight at ≤ 20 weeks (mean 16.2 ± weeks) women would have experienced some weight gain could have potentially influenced gestational weight gain estimate. Therefore, GWG rate used in this study was a more appropriate measure as recommended by IOM guidelines [1].*

3. Additionally, while the authors responded that the findings presented in Table 3 are crude relative risks, it is essential that this be clearly indicated in the manuscript, including within the table itself.

- Unadjusted indicated in Table 3 and its description

4. In Table 2, the authors calculated odds ratios to assess factors associated with insufficient or excessive gestational weight gain. However, they interpreted these findings as if they were relative risks. They used the term "likelihood," which is not appropriate when discussing odds ratios.

- Likelihood is replaced with odds throughout the manuscript

1.	IOM. Weight Gain During Pregnancy: Reexamining the Guidelines

In: Rasmussen KM, Yaktine AL, editors. The National Academies Collection: Reports funded by National Institutes of Health. Washington (DC): National Academies Press (US)

Copyright © 2009, National Academy of Sciences.; 2009.

2.	Malik VS, Schulze MB, Hu FB. Intake of sugar-sweetened beverages and weight gain: a systematic review. The American journal of clinical nutrition. 2006;84(2):274-88.

3.	Luger M, Lafontan M, Bes-Rastrollo M, Winzer E, Yumuk V, Farpour-Lambert N. Sugar-Sweetened Beverages and Weight Gain in Children and Adults: A Systematic Review from 2013 to 2015 and a Comparison with Previous Studies. Obesity facts. 2017;10(6):674-93.

4.	Maslova E, Halldorsson TI, Astrup A, Olsen SF. Dietary protein-to-carbohydrate ratio and added sugar as determinants of excessive gestational weight gain: a prospective cohort study. BMJ open. 2015;5(2):e005839.

5.	Malik VS, Hu FB. The role of sugar-sweetened beverages in the global epidemics of obesity and chronic diseases. Nature Reviews Endocrinology. 2022;18(4):205-18.

6.	Fishman E, Böcker L, Helbich M. Adult Active Transport in the Netherlands: An Analysis of Its Contribution to Physical Activity Requirements. PloS one. 2015;10(4):e0121871.

7.	Wanner M, Götschi T, Martin-Diener E, Kahlmeier S, Martin BW. Active transport, physical activity, and body weight in adults: a systematic review. American journal of preventive medicine. 2012;42(5):493-502.

8.	WHO. Physical activity. World Health Organization. 2016.

9.	Chasan-Taber L, Schmidt MD, Roberts DE, Hosmer D, Markenson G, Freedson PS. Development and validation of a Pregnancy Physical Activity Questionnaire. Medicine and science in sports and exercise. 2004;36(10):1750-60.

10.	Chasan-Taber L, Silveira M, Pekow P, Braun B, Manson JE, Solomon CG, Markenson G. Physical activity, sedentary behavior and risk of hypertensive disorders of pregnancy in Hispanic women. Hypertension in pregnancy. 2015;34(1):1-16.

11.	Adeniyi AF, Ogwumike OO. Physical activity and energy expenditure: findings from the Ibadan Pregnant Women's Survey. African journal of reproductive health. 2014;18(2):117-26.

12.	Adeoye IA. Pattern and correlates of physical activity and sedentary behaviours of pregnant women in Ibadan, Nigeria: Findings from Ibadan pregnancy cohort study. PLOS Global Public Health. 2022;2(10):e0001153.

13.	Baker JH, Rothenberger SD, Kline CE, Okun ML. Exercise during early pregnancy is associated with greater sleep continuity. Behavioral sleep medicine. 2018;16(5):482-93.

14.	da Silva SG, Ricardo LI, Evenson KR, Hallal PC. Leisure-time physical activity in pregnancy and maternal-child health: a systematic review and meta-analysis of randomized controlled trials and cohort studies. Sports medicine. 2017;47(2):295-317.

15.	Russo LM, Nobles C, Ertel KA, Chasan-Taber L, Whitcomb BW. Physical activity interventions in pregnancy and risk of gestational diabetes mellitus: a systematic review and meta-analysis. Obstetrics & Gynecology. 2015;125(3):576-82.

16.	Bhattacharya S, Campbell DM, Liston WA, Bhattacharya S. Effect of Body Mass Index on pregnancy outcomes in nulliparous women delivering singleton babies. BMC public health. 2007;7(1):168.

17.	Sebire NJ, Jolly M, Harris JP, Wadsworth J, Joffe M, Beard RW, Regan L, Robinson S. Maternal obesity and pregnancy outcome: a study of 287,213 pregnancies in London. International journal of obesity and related metabolic disorders : journal of the International Association for the Study of Obesity. 2001;25(8):1175-82.

18.	Fyfe EM, Thompson JMD, Anderson NH, Groom KM, McCowan LM. Maternal obesity and postpartum haemorrhage after vaginal and caesarean delivery among nulliparous women at term: a retrospective cohort study. BMC pregnancy and childbirth. 2012;12(1):112.

19.	Butwick AJ, Abreo A, Bateman BT, Lee HC, El-Sayed YY, Stephansson O, Flood P. Effect of Maternal Body Mass Index on Postpartum Hemorrhage. Anesthesiology. 2018;128(4):774-83.

20.	Blomberg M. Maternal Obesity and Risk of Postpartum Hemorrhage. Obstetrics & Gynecology. 2011;118(3):561-8.

21.	Siega-Riz AM, Bodnar LM, Stotland NE, Stang. The Current Understanding of Gestational Weight Gain among Women with Obesity and the Need for Future Research. NAM Perspectives Discussion Paper,. National Academy of Medicine, Washington, DC. 2019.

22.	Institute of M, National Research Council Committee to Reexamine IOMPWG. The National Academies Collection: Reports funded by National Institutes of Health. In: Rasmussen KM, Yaktine AL, editors. Weight Gain During Pregnancy: Reexamining the Guidelines. Washington (DC): National Academies Press (US)

Copyright © 2009, National Academy of Sciences.; 2009.

23.	Drehmer M, Duncan BB, Kac G, Schmidt MI. Association of second and third trimester weight gain in pregnancy with maternal and fetal outcomes. PloS one. 2013;8(1):e54704.

24.	Campos CAS, Malta MB, Neves PAR, Lourenço BH, Castro MC, Cardoso MA. Gestational weight gain, nutritional status and blood pressure in pregnant women. Revista de saude publica. 2019;53:57.
