# Supplementary material for: Gestational weight gain among pregnant women in Ibadan, Nigeria: Pattern, predictors and pregnancy outcomes
Source: PLoS One. 2023 Aug 18;18(8):e0290102. doi: 10.1371/journal.pone.0290102 (PMC10437817; doi:10.1371/journal.pone.0290102)
Supplement: Attachment — Submitted filename: Reviewer comments.docx [file pone.0290102.s001.docx]

This study determines factors affecting gestational weight gain and its association with maternal outcomes in Nigeria. Please find below my individual comments.

Abstract

The results did not reflect all findings from the study, e.g., findings from Table 4

Introduction

1. Overall, the introduction section is slightly general without the support of facts or evidence, such as percentages or rates. This information is important to support the study's rationale and for the reader to understand the actual situation of problems. Suggest including some background information on the GWG pattern in Nigeria and other related regions
   a. Line 124-129: Suggest elaborate these statements, e.g., What is the percentage of excessive and insufficient in the countries?
   b. Line 129-131: please elaborate

Figure: Suggest replacing Figure 1 with the flow chart of study participants, whereby how the study participants were selected.

Materials and Methods:

2. Line 164: …at investing.., suggest …to investigate..
3. Line 215- 254: Since lifestyle information was collected in this study, especially the sugar-sweetened beverages, and sleep pattern, and physical activity (line 178-180), wondering why the authors did not investigate the associations between lifestyle factors and GWG. This will strengthen the value of the article. Suggest state clearly how the variables were selected to be included in the model
4. Line 256: please list out the adjusted variables
5. Line 209: please include the source of reference

Results:

6. Line 315: “'20,000-99,999', please provide a footnote on the rate of USD for ease of interpretation

Discussion:

7. Line 406: .. had appropriate weight.., suggest normal GWG
8. Line 423-430: Since the authors noticed that maternal lifestyle behaviours, such as nutrition and physical activity, are important factors contributing to GWG, suggesting the author included these factors in the analysis (Table 2)
9. Line 451-455: please include the source of reference; what is the recommended GWG practice in Nigeria? Suggest including this information in the introduction
10. Line 472-474: Any information on the types of cesarean sections performed? Elective or emergency cesarean section
